# Delineation of two multi-invasion-induced rearrangement pathways that differently affect genome stability

**DOI:** 10.1101/gad.350618.123

**Published:** 2023-07-01

**Authors:** Diedre Reitz, Yasmina Djeghmoum, Ruth A. Watson, Pallavi Rajput, Juan Lucas Argueso, Wolf-Dietrich Heyer, Aurèle Piazza

**Affiliations:** 1 Department of Microbiology and Molecular Genetics, University of California, Davis, Davis, California 95616, USA;; 2 Laboratory of Biology and Modelling of the Cell (UMR5239), Ecole Normale Supérieure de Lyon, 69007 Lyon, France;; 3 Department of Environmental and Radiological Health Sciences, Colorado State University, Fort Collins, Colorado 80523, USA;; 4 Department of Molecular and Cellular Biology, University of California, Davis, Davis, California 95616, USA

**Keywords:** DNA break, DNA joint molecules, genomic instability, polymerase *δ*, homologous recombination

## Abstract

Punctuated bursts of structural genomic variations (SVs) have been described in various organisms, but their etiology remains incompletely understood. Homologous recombination (HR) is a template-guided mechanism of repair of DNA double-strand breaks and stalled or collapsed replication forks. We recently identified a DNA break amplification and genome rearrangement pathway originating from the endonucleolytic processing of a multi-invasion (MI) DNA joint molecule formed during HR. Genome-wide approaches confirmed that multi-invasion-induced rearrangement (MIR) frequently leads to several repeat-mediated SVs and aneuploidies. Using molecular and genetic analysis and a novel, highly sensitive proximity ligation-based assay for chromosomal rearrangement quantification, we further delineate two MIR subpathways. MIR1 is a universal pathway occurring in any sequence context, which generates secondary breaks and frequently leads to additional SVs. MIR2 occurs only if recombining donors exhibit substantial homology and results in sequence insertion without additional breaks or SVs. The most detrimental MIR1 pathway occurs late on a subset of persisting DNA joint molecules in a PCNA/Pol*δ*-independent manner, unlike recombinational DNA synthesis. This work provides a refined mechanistic understanding of these HR-based SV formation pathways and shows that complex repeat-mediated SVs can occur without displacement DNA synthesis. Sequence signatures for inferring MIR1 from long-read data are proposed.

## Abstract (precis)

In this study, Reitz et al. delineate two molecular models for multi-invasion-induced rearrangements (MIRs) involved in homologous recombination. Using the *S. cerevisiae* LYS2 half-locus experimental system and a novel method for detecting rare translocation events, they provide mechanistic insight into the kinetics and dependencies of MIRs during chromosomal segregation and the formation of structural variations.

Structural variants (SVs) of the genome are the predominant class of driver mutations in most cancer types and are more numerous than the more widely studied single-nucleotide variants and indels (The ICGC/TCGA Pan-Cancer Analysis of Whole Genomes Consortium 2020; Cosenza et al. 2022). These SVs fuel cancer onset and its evolution, promoting metastasis and chemoresistance (Notta et al. 2016). Clusters of SVs typical of chromothripsis were found in >20% of 2658 patients in a pan-cancer analysis and often play a causal role in cancer development (Notta et al. 2016; The ICGC/TCGA Pan-Cancer Analysis of Whole Genomes Consortium 2020). The molecular processes underlying these complex SVs are incompletely characterized (Cosenza et al. 2022).

The maintenance of genome stability involves independent molecular mechanisms that inhibit the occurrence of DNA damage and achieve their accurate repair (Putnam and Kolodner 2017). Homologous recombination (HR) is a universal DNA damage repair and tolerance mechanism that uses an intact homologous dsDNA as a template. As such, it is often considered a high-fidelity repair mechanism. However, extensive experimental evidence has implicated HR in the formation of repeat-mediated SVs, an outcome exacerbated in various mutant contexts (Putnam and Kolodner 2017; Savocco and Piazza 2021). These SVs originate either (1) from unrestricted DNA synthesis initiated at an ectopic (i.e., nonallelic) displacement loop (D-loop) DNA joint molecule (JM) in a process called break-induced replication (BIR) or (2) by endonucleolytic processing of ectopic JMs generated throughout the pathway (Savocco and Piazza 2021).

The tenet of BIR is a displacement DNA synthesis step that goes unabated until stabilization of the DSB end by either telomere capture, merging with a converging replication fork, D-loop disruption and annealing of the extended ssDNA to a complementary DNA end, or end joining (Anand et al. 2013). BIR depends on processive displacement DNA synthesis by Polδ-PCNA in the context of a D-loop, which is reduced in the absence of Pol32 (Lydeard et al. 2007; Payen et al. 2008; Li et al. 2009; Brocas et al. 2010; Donnianni and Symington 2013; Saini et al. 2013; Donnianni et al. 2019). Consequences of BIR range from loss of heterozygosity to nonreciprocal translocation associated with a copy number gain upon allelic and ectopic donor usage, respectively. More complex rearrangements may result from frequent template switches over the course of BIR (Smith et al. 2007; Ruiz et al. 2009; Anand et al. 2014). BIR is thus a HR-based mechanism that can generate complex, neosynthesized chromosomal rearrangements from a single DSB end.

The role of structure-selective endonucleases (SSEs) in the generation of HR-mediated chromosomal rearrangements has long been recognized as part of the canonical double-strand break repair (DSBR) model. Endonucleolytic resolution of an allelic interhomolog double-Holliday junction (dHJ) intermediate can lead to the formation of either a crossover or a noncrossover with equal likelihood. Resolution into a crossover can cause loss of heterozygosity of the centromere-devoid chromosomal region at the next cell generation (Szostak et al. 1983). If DNA strand invasion occurs at an ectopic repeat, a crossover can lead to various types of repeat-mediated SVs, including insertions, deletions, inversions, and translocations (Argueso et al. 2008; Sampaio et al. 2020). This vulnerability is not restricted to the resolution of dHJs, as other upstream JMs such as D-loops can also be processed by SSEs (Deem et al. 2008; Ruiz et al. 2009; Smith et al. 2009; Mazón et al. 2012; Pardo and Aguilera 2012; Mazón and Symington 2013).

We previously identified a genomic instability mechanism originating from “multi-invasions” (MIs), a JM in which a single DSB end has invaded two independent dsDNA donors (Fig. 1A; Piazza et al. 2017). MIs are readily formed in reconstituted in vitro reactions with ssDNA substrates of physiological length (i.e., >100 nt) and yeast and human Rad51, Rad54, and RPA proteins, as well as in *Saccharomyces cerevisiae* cells (Wright and Heyer 2014; Piazza et al. 2017, 2021b). They are presumably by-products of homology search by intersegmental contact sampling demonstrated in vitro for RecA (Forget and Kowalczykowski 2012). This tripartite recombination mechanism, termed multi-invasion-induced rearrangement (MIR), generates an oriented translocation between the two engaged donors in a HR- and SSE-dependent fashion (Piazza et al. 2017). MIR inserts the intervening sequence of the invading molecule between the two translocated donors. It occurs at varying frequencies depending on homology length and spatial location of the recombining partners, reaching frequencies of up to 1% in wild-type cells. Depending on the sequence context, it yields additional rearrangements between the DSB and donor molecules at high frequencies from 15% to 85% of MIR recombinants (see the genome-wide approaches below; Piazza et al. 2017). Finally, MIR relies only partially on Pol32, suggesting that at least a fraction of MIR events and/or the repair of newly generated breaks does not require extensive displacement DNA synthesis. Thus, MIR may generate punctuated bursts of SVs with no or limited DNA synthesis involved, unlike BIR.

Here we investigated the existence of two MIR subpathways postulated on the basis of past molecular and genetic evidence: MIR1 that generates a translocation and additional DSB ends, applicable in any sequence context, and MIR2 that generates an insertion without forming an additional DSB but has specific sequence requirements (see below; Fig. 2A; Piazza et al. 2017; Piazza and Heyer 2018). We devised a highly sensitive molecular assay to quantify specific chromosomal rearrangements, which revealed that MIR1 occurs late on persistent DNA JMs enriched for MIs. Importantly, MIR1 occurs in the absence of Pol*δ*-PCNA, unlike D-loop extension. These results elucidate a novel HR-based mechanism leading to tripartite SVs in the absence of displacement DNA synthesis.

## Results

### Experimental system

**Figure 1.**
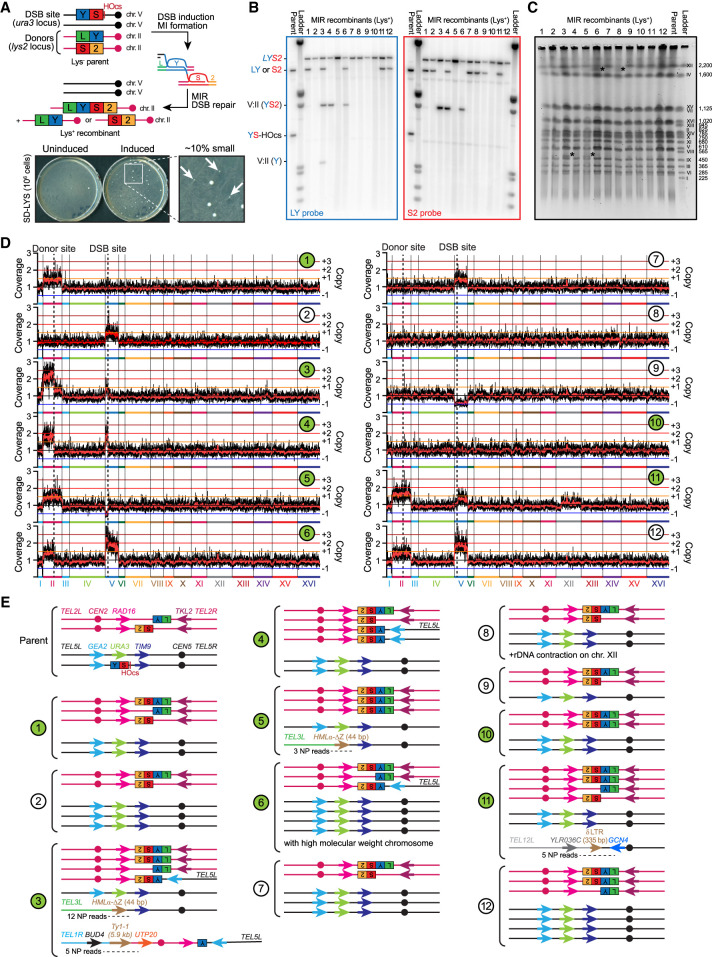
MIR frequently causes additional unselected rearrangements. (*A*) Experimental system in diploid *S. cerevisiae*. The heterozygous DSB-inducible *YS*-HOcs construct replaces *URA3* on chromosome V. The *LY* and *S2* donors consist in the two halves of the *LYS2* gene (2090 and 2089 bp, respectively) at its locus on chromosome II. This DSB donor configuration is referred to as interchromosomal with allelic donors. Translocation of the *LY* and *S2* donors restores a functional *LYS2* gene. Approximately 10% of induced Lys^+^ colonies are small on the initial plate and exhibit a higher proportion of additional SVs than bigger colonies. (*B*–*D*) Twelve small MIR recombinants were analyzed by Southern blot (*B*), PFGE (*C*), and high-throughput shotgun sequencing (*D*). Strains labeled in green were additionally analyzed by aCGH and nanopore long-read sequencing. (*C*) The ladder corresponds to a *S. cerevisiae* strain from the YPH80 background, marginally different from our W303 parental strain. Ladder size is in kilobases. Chromosome V and chromosome VIII comigrate in the W303 background. (*) Chromosomal abnormality. (*E*) Deduced genome structure of the 12 MIR recombinants. The number of nanopore (NP) reads encompassing the unselected rearrangements is indicated.

We previously developed an experimental system in diploid *S. cerevisiae* cells that specifically selects the MIR translocation product (Piazza et al. 2017, 2021b). This product, formed by a rearrangement that unites the two halves of the *LYS2* gene, leads to lysine prototrophy (Lys^+^ colonies). The system has three components: a site-specific DSB-inducible construct and two donors. The heterozygous DSB-inducible construct on chromosome V consists of the HO cut site (HOcs) and, on one side, the central part (*YS*) of the *LYS2* gene. This *YS* sequence has nonoverlapping homology with two donors: the LY and S2 halves of the *LYS2* gene (Fig. 1A). The Y and S shared homologies are ∼1 kb long each (Fig. 1A). In the reference strain used previously (Piazza et al. 2017), these donors were located at an allelic position on chromosome II, in place of the original *LYS2* gene. In this heterozygous donor configuration, referred to as “allelic,” the sequences flanking the donors were identical. Other configurations were also used in which the two donors were located at different, ectopic loci. These donor configurations are referred to as “ectopic,” with donors devoid of flanking homologies (see below). These latter configurations resulted in higher frequencies of secondary rearrangements (Piazza et al. 2017).

DSB formation at the HOcs is achieved with ∼99% efficiency within 1 h of induction of *HO* gene expression (Piazza et al. 2017, 2021b). Its repair by gene conversion off the homolog takes up to 6 h, during which MIR can occur (Fig. 1A; Piazza et al. 2021a,b). The basal and induced frequencies of Lys^+^ cells were determined for multiple independent cultures by plating cells on selective media prior to and 2 h after DSB induction, respectively (Fig. 1A). In our reference wild-type strain with the three components of the system (i.e., the DSB site and the two donors) on different chromosomes, DSB induction in liquid media caused a 100-fold increase in the frequency of Lys^+^ colonies, at 3.08 × 10^−5^ ± 0.3 × 10^−5^ colonies (see below; Piazza et al. 2017). MIR can be orders of magnitude more frequent with donors on the same chromosomes (Piazza et al. 2017).

### MIR frequently causes additional unselected rearrangements

Southern blot and quantitative PCR (qPCR) analysis of MIR products confirmed that all recombinants resulted in the formation of the *LYS2* gene at its expected locus, segregating with either the *LY* or *S2* donor. In a typical recombinant, the inducible DSB site is lost through gene conversion off the homologous chromosome V (Fig. 1A; Piazza et al. 2017). In ∼15% of total recombinants, additional, unselected rearrangements between the *Y-*containing and *S-*containing parts of our experimental system were observed in addition to the MIR product (Piazza et al. 2017). The prevalence of these additional rearrangements was much higher (∼90%) in small Lys^+^ colonies, which represent ∼10% of the recombinants (Fig. 1A). Most (15 out of 20) colonies appearing small on the selection plate did not exhibit a growth defect upon restreaking (Supplemental Fig. S1A). Consequently, their size reflected mainly a delayed onset of growth resumption following DSB formation. Here, we sought to determine MIR-associated SVs and copy number variation (CNV) genome-wide. To this end, 12 small Lys^+^ recombinants were analyzed by Southern blot (Fig. 1B), pulse-field gel electrophoresis (PFGE) (Fig. 1C), and paired-end short-read high-throughput sequencing (Fig. 1D). A subset (#1, #3, #4, #5, #6, #10, and #11) was further analyzed by array comparative genome hybridization (aCGH) and long-read nanopore sequencing. The structure of the recombinant chromosomes is depicted in Figure 1E.

The *LYS2* gene was restored at its locus in all (12 out of 12) cases, as expected (Fig. 1B). This translocation segregated either with the *LY* or the *S2* donor in eight out of 12 cases (Fig. 1B). In two out of 12 cases (#5 and #10) no remaining donor was visible, while in the last two out of 12 cases (#1 and #11) the *LY* and *S2* donors were both present together with the *LYS2* translocation (Fig. 1B). Finally, four additional SVs involving chromosome II and chromosome V were visible in three out of 12 recombinants (#3, #4, and #6). In total, six out of 12 colonies (#1, #3, #4, #5, #6, and #10) exhibited an additional chromosomal abnormality involving the DSB and/or the donors visible by Southern blot. Chromosome length determination by PFGE further revealed unambiguous chromosome abnormalities in four out of 12 recombinants, in the form of either an ∼470-kb band (#3 and #5) or an ∼2-Mb band (#6 and #8) (Fig. 1C). Hence, seven out of 12 small Lys^+^ recombinants exhibited a detectable additional chromosome II and/or chromosome V abnormality together with the MIR translocation, consistent with past findings (Piazza et al. 2017).

High-throughput sequencing further revealed that all (12 out of 12) of the analyzed small Lys^+^ MIR recombinants bore at least one additional chromosomal abnormality in addition to the selected MIR translocation. A total of 13 whole-chromosome aneuploidies was detected in nine out of 12 strains analyzed, all of which involved chromosome II and chromosome V (Fig. 1D). The majority of additional SVs (five out of nine SVs in three out of four strains containing additional SVs) involved the *YS* break site on chromosome II and/or chromosome V (Fig. 1D). However, in a substantial number of cases (four out of nine SVs in three out of four strains), other chromosomes were also found to be involved, together with chromosome II and/or chromosome V. In colony #11, a translocation involving the right arm of chromosome XII and the left arm of chromosome V led to a 1296-kb-long chromosome. The SV occurred between two ∼335-bp Solo *δ* elements sharing 89% sequence similarity: *YELWδ6* from chromosome V and *YLRCδ3* from chromosome XII (Fig. 1E). Colonies #3 and #5 exhibited a translocation between the right side of the DSB site on chromosome V and the left side of *HMLα* on chromosome III, yielding a 474-kb chromosome visible by PFGE (Fig. 1C). The SV occurred at the 44-bp homology between the right side of the HO cut site (Fig. 1E). Finally, colony #3 exhibited an additional translocation between the right arm of chromosome I and the left arm of chromosome II. This latter translocation occurred at the position of two ∼5.9-kb Ty1-1 retrotransposons sharing 92% homology: *YARCTy1-1* on chromosome I and *YBLWTy1-1* on chromosome II. The resulting I:II translocated chromosome is expected to be 655 kb long but was not observed by PFGE (Fig. 1C). Consequently, we favor the alternative possibility that the translocation occurred on the chromosome II arm already involved in a chromosome II:V translocation, yielding a I:II:V translocated chromosome of 433 kb migrating roughly at the same position as chromosome IX (Fig. 1E). In all cases, substantial homologies (44 bp, 335 bp, and 5.9 kb) were present at these SV junctions. Finally, a distinct ∼2-Mb chromosome is visible by PFGE in recombinants #6 and #8 (Fig. 1C). Recombinant #6 bears the highest copy number gain of chromosome V of all recombinants (Fig. 1D), which may give rise to this variant chromosome. Recombinant #8 does not exhibit chromosomal copy number change (Fig. 1D), but the ribosomal DNA (rDNA) coverage is reduced by ∼40% relative to the parental strain (Supplemental Fig. S1B). This band may thus correspond to a large heterozygous rDNA contraction on chromosome XII.

In conclusion, detailed characterization of a subset of the ∼10% of small MIR recombinants confirmed that MIR is frequently associated with secondary rearrangements in addition to the primary selected translocation event (Piazza et al. 2017). These rearrangements are specific to MIR cells, as no such rearrangements were observed following HO-induced DSB repair that did not select for MIR events (Sakofsky et al. 2014). It also revealed the involvement of other chromosomes together with those initiating MIR at sites of extensive homologies, suggesting a HR-dependent origin for these co-occurring rearrangements. The high frequency of whole-chromosome aneuploidies also indicated that MIR is associated with chromosome missegregation. We sought to better characterize the MIR mechanism(s) leading to the formation of these secondary rearrangements.

### Existence of two MIR subpathways with different requirements for displacement DNA synthesis

**Figure 2.**
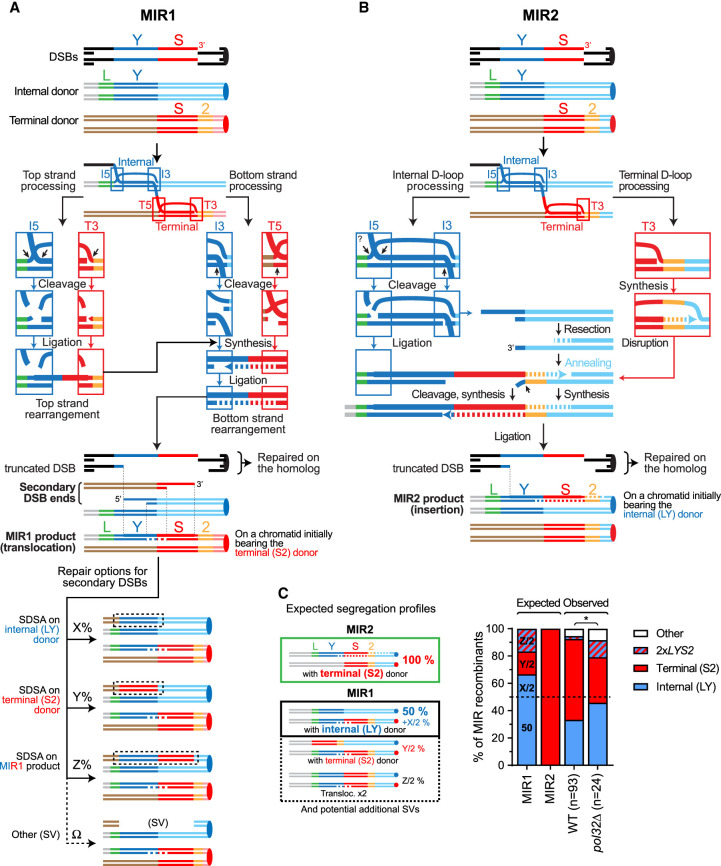
Models of multi-invasion-induced rearrangements. (*A*) MIR1 model. MIR1 generates a translocation and two additional single-ended DSBs. (*B*) MIR2 model. MIR2 generates an insertion. (*C*) Segregation products expected from MIR1 and MIR2 and products observed in normal-sized Lys^+^ colonies in wild-type (APY89) and *pol32*Δ (WDHY4408) backgrounds. Wild-type data are from Southern blot presented in Piazza et al. (2017). Southern blot images for *pol32*Δ are shown in Supplemental Figure S2C. “Other” refers to strains bearing no donor and/or an SV involving one donor and the initiating DSB region. (*) *P* < 0.05, Fisher's exact test.

We postulated the existence of two MIR pathways, MIR1 and MIR2, to account for (1) the segregation pattern of the *LY* and *S2* donors together with the MIR translocation and (2) the presence of additional chromosomal rearrangements (Fig. 2; Supplemental Fig. S2A; Piazza and Heyer 2018). In the fully endonucleolytic MIR1 pathway, all four JM junctions are cleaved by SSEs (Fig. 2A). This pathway yields two additional one-ended DSBs, in addition to the translocation carried in place of the terminal *S2* donor (Fig. 2A). In an allelic donor configuration, repair of these secondary DSBs will yield an *LY*, an *S2*, or a second MIR product (*LYS2*), depending on the template used (Fig. 2A; detailed in Supplemental Fig. S2A). This recombination product or the intact sister chromatid (internal *LY* donor) will segregate with the primary MIR translocation at the next cell division. If the donors are in an ectopic configuration, accurate repair is not possible, and additional rearrangements are observed at high frequencies (see below; Piazza et al. 2017).

In contrast, MIR2 resolves the terminal invasion by synthesis-dependent strand annealing (SDSA), in which the extended end of the terminal D-loop pairs with the single-ended DSB generated upon cleavage of the internal D-loop (Fig. 2B). This pathway yields an insertion without additional DSBs. Consequently, the MIR2 product will segregate 100% of the time with the terminal *S2* donor. No secondary rearrangement is expected.

The MIR1 and MIR2 mechanisms thus make clear predictions as to which donor segregates with the MIR translocation product (Fig. 2C). The observed segregation pattern, however, does not satisfy any single prediction (Fig. 2C), suggesting that both MIR1 and MIR2 mechanisms are at play with donors in an allelic configuration in wild-type cells (Fig. 1A). Although the MIR1 and MIR2 pathways have largely overlapping requirements (HR and SSE), they are expected to differ in the following ways: (1) The processing of the terminal JM in the MI differs: It is cleaved in MIR1 and extended in MIR2. Moreover, the top strand of a MIR1 translocation can theoretically be produced without DNA synthesis (Fig. 2A). (2) Their reliance on displacement DNA synthesis and 3′ homologies between the two donors differs, as displacement DNA synthesis is required only for MIR2 (Fig. 2B). (3) A feature specific to MIR1 is that it generates two additional DSBs, which have the potential to lead to secondary rearrangements.

In the following sections, we address the existence of the MIR1 and MIR2 pathways by challenging these predictions and better define their relative contributions to the formation of chromosomal rearrangements in different sequence contexts.

### The absence of Pol32 causes a shift from the MIR2 to the MIR1 donor segregation profile

In order to gain insight into the mechanisms at play in the allelic donor situation and address the prediction that MIR2 requires displacement DNA synthesis, we analyzed MIR recombinants obtained in a *pol32*Δ mutant defective for long-range recombination-associated DNA synthesis. *POL32* deletion caused a significant 2.5-fold decrease in MIR frequency (Supplemental Fig. S2B; Piazza et al. 2017). Southern blot analysis of 24 recombinants revealed a significant shift in the donor segregation pattern (Fig. 2C; Supplemental Fig. S2C). Notably, the expected MIR2 signature (*LYS2* + *S2*) decreased from 55 out of 93 to eight out of 24 (*P* = 0.037, Fisher's exact test), with a compensatory increase in MIR1 signatures (*LYS2* + *LY* and 2× *LYS2*). The proportion of *LYS2* + *LY* recombinants reached 45.8%, approaching the minimal proportion expected for MIR1-only events (50%) (Fig. 2C). The share of strains exhibiting additional chromosomal abnormalities also increased, as expected for a larger fraction of MIR1 events. These results are consistent with our inference that both MIR1 and MIR2 contribute to MIR translocations. They support our first prediction that MIR2 relies on displacement DNA synthesis. However, because significant displacement DNA synthesis can occur in the absence of *POL32* (McVey et al. 2016), it was unclear from this analysis whether MIR1 also requires displacement synthesis (see below).

### MIR2, but not MIR1, requires long homologies downstream from the donors

**Figure 3.**
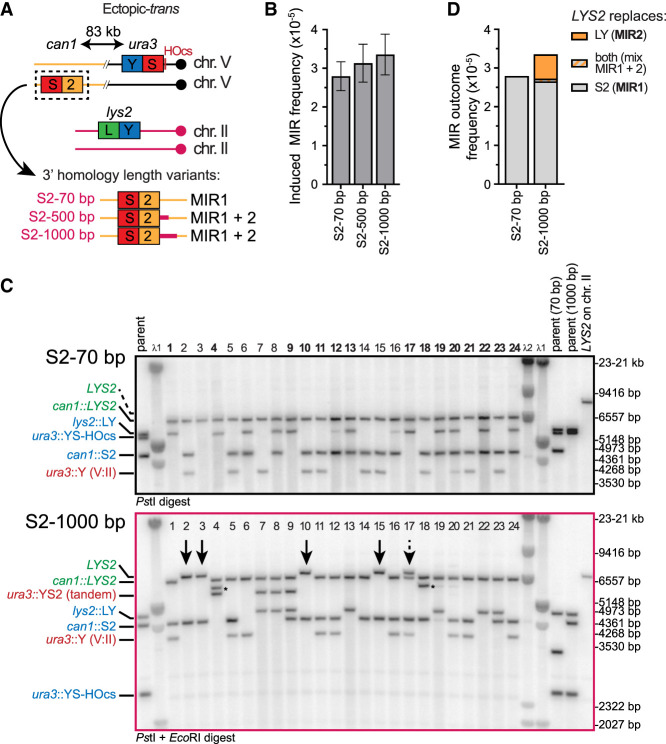
MIR2 uniquely requires homology at the 3′ side of each donor. (*A*) Experimental system to address the existence of MIR1 and MIR2 mechanisms. The *LY* and *S2* donors are ectopically located. The *S2* donor is in *trans* relative to the DSB site on the chromosome V homolog. Homology with the 3′ flanking sequence of the *LY* donor is increased near the *S2* donor, from 70 to 1000 bp (purple region), corresponding to the chromosome II sequence. This extension does not contain full-length open reading frames. (*B*) MIR frequency in wild-type cells bearing 70, 500, and 1000 bp of homology 3′ of the LY and S2 donors (APY85, APY86, and APY87, respectively). (*C*) Southern blot analysis of 24 Lys^+^ recombinants obtained with either 70 bp (*n* = 46) or 1000 bp (*n* = 48) of 3′ homology. For details on substrates and product lengths, see Supplemental Figure S3A. Remaining colonies are presented in Supplemental Figure S3B. Arrows indicate MIR2 products. The dashed arrow indicates a mixed colony. (*) Band of unknown origin. (*D*) MIR outcome frequencies.

Our models posit that MIR2, but not MIR1, requires homology flanking the donors at their 3′ side. To address this specific prediction, we moved the terminal (*S2*) donor to an ectopic location on chromosome V (*CAN1* locus, 83 kb away from the DSB site at *URA3*) (see Fig. 3A; Piazza et al. 2017). In this configuration, referred to as “ectopic *trans*,” the donors do not share extensive flanking homologies (only the 70-bp *LYS2* terminator), unlike in the initial allelic configuration. Only MIR1 is expected in this configuration, whose outcome is a chromosome II:V translocation yielding an ∼895-kb chromosome with *LYS2* present in place of the *S2* donor at *CAN1*. We then restored substantial flanking homologies (500 and 1000 bp, from the initial 70 bp) at the 3′ side of the donors by modifying the *S2* donor (Fig. 3A). Restoration of this 3′ flanking homologous region is expected to enable MIR2, whose outcome is *LYS2* in place of the *LY* donor without associated translocation. Hence, MIR1 and MIR2 products can be straightforwardly distinguished.

Addition of flanking homology 3′ of the *S2* donor led to a modest, nonsignificant increase in MIR frequency (Fig. 3B). We determined the structure of the recombinants by Southern blot analysis on 46 and 48 Lys^+^ colonies obtained with the *S2* 70-bp and the *S2* 1000-bp donor constructs, respectively (Fig. 3C; Supplemental Fig. S3B). Different restriction digestions were performed with each construct to be able to unambiguously distinguish parental molecules as well as primary and secondary rearrangement products (see Supplemental Fig. S3A for a description of the various products). With the *S2* 70-bp construct, all *LYS2* products were in the form of a chromosome II:V translocation that replaced the *S2* donor at *CAN1*, as expected for a MIR1-only mechanism (Fig. 3C; Supplemental Fig. S2B). PFGE analysis of a subset of 24 recombinants confirmed the presence of an ∼900- to 930-kb neochromosome in all cases (Supplemental Fig. S3C,D), consistent with the length expected for such a chromosome II:V translocation (∼915 kb) (see more below).

With the *S2* 1000-bp construct, however, *LYS2* was found in place of the *LY* donor on chromosome II in nine out of 48 cases, as expected for MIR2 products (arrows in Fig. 3C; Supplemental Fig. S2B), significantly different from the zero out of 46 observed with the *S2* 70-bp donor (*P* = 2.5 × 10^−3^, two-tailed Fisher's exact test). Of the 48 remaining colonies, 38 had *LYS2* in place of the *S2* donor, typical of MIR1, and one was a mixed colony (Fig. 3C; Supplemental Fig. S2B). Thus, providing 3′ flanking homology between the donors is sufficient to restore MIR2. Combining these frequencies with total MIR frequency data indicates that these MIR2 events did not occur at the expense of MIR1 events but were in addition to them, at an approximately fivefold lower frequency than MIR1 events (∼5 × 10^−6^ vs. 2.7 × 10^−5^, respectively) (Fig. 3D). These observations corroborate our second prediction that MIR2, but not MIR1, requires substantial homology between the donors’ 3′ flanking sequences.

### MIR1, but not MIR2, is frequently associated with secondary rearrangements

**Figure 4.**
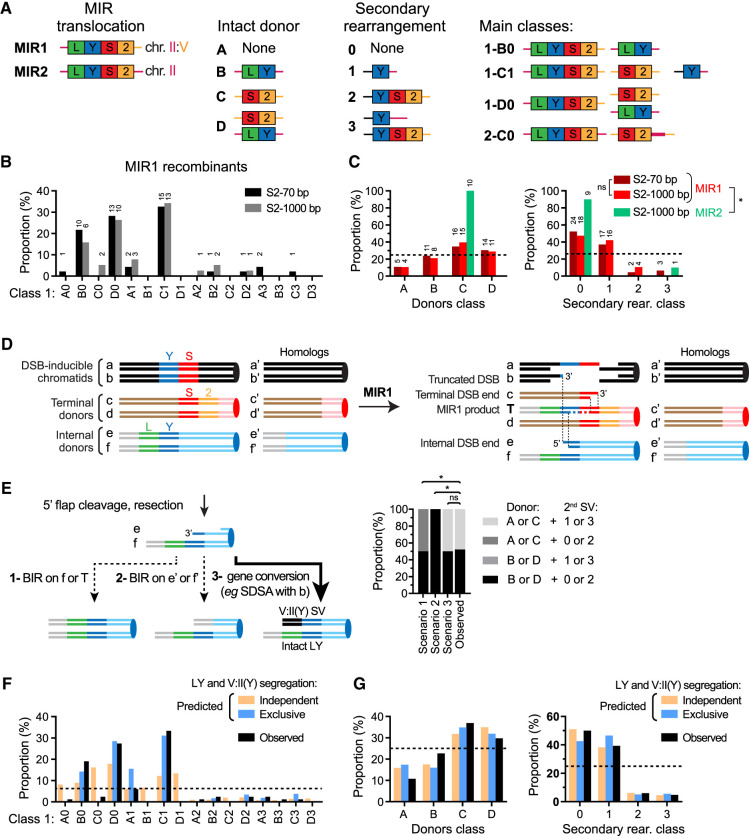
MIR1 is frequently associated with additional structural rearrangements, unlike MIR2. (*A*) Classification of Lys^+^ recombinants based on MIR type, remaining donor(s), and additional rearrangement involving the donor and the break site. The most frequent MIR classes are depicted. (*B*) Distribution of MIR1 events obtained with the *S2* 70-bp (*n* = 46) and *S2* 1000-bp (*n* = 38) donor constructs. (*C*) Distribution of donor segregation (*left*) and secondary rearrangements (*right*) in MIR1 and MIR2 events obtained with the *S2* 70-bp and *S2* 1000-bp donor constructs. Number of events is indicated *above* each bar in *B* and *C*. (*D*) Expected location of the single-ended DSBs generated on the internal (*LY*) and terminal (*S2*) donors. Each chromatid is labeled. Note that in the ectopic *trans* donor configuration, a/b are equivalent to c′/d′, and c/d are equivalent to a′/b′. They are not shown connected for the sake of simplicity and to avoid inducing uncertain associations between the repair of the initiating DSB ends and of the secondary DSB generated at the terminal donor. (*E*, *left*) Three scenarios for the repair of the single-ended DSB at the internal (LY) donor. (*Right*) The expected donor and SV classes for each scenario, and the observed class distributions. (*) *P* < 0.05, χ^2^ contingency test. (*F*) Predicted MIR1 class distribution assuming either independent or exclusive segregation of the *LY* donor and the V:II(Y) SV and observed class distribution of the pooled *S2* 70-bp-containing and *S2* 1000-bp-containing strains (*n* = 84). (*G*) Same as *F*, with distribution pooled by class (*left*) and secondary rearrangements (*right*).

MIR recombinants were subclassified based on their category (MIR1 or MIR2), the segregating donor(s) (A–D), and the secondary rearrangements detected (0–3) to yield classes in the form of, for example, 1-C1 (Fig. 4A). First, MIR1 events obtained with *S2* 70 bp and *S2* 1000 bp did not exhibit significant differences in their class distribution (Fig. 4B). Half the recombinants exhibited at least one secondary chromosome II:V translocation, and 39%–41% exhibited a donor CNV (class A or D) (Fig. 4B). In total, 78.3% and 78.9% of MIR1 recombinants originating from *S2* 70-bp-containing or *S2* 1000-bp-containing strains, respectively, exhibited at least one additional chromosomal abnormality (Piazza et al. 2017). In contrast, only one out of nine MIR2 translocants (#33) contained an additional SV (Supplemental Fig. S3B). This significant difference (*P* < 3 × 10^−4^ in both cases, two-tailed Fisher's exact test) fulfills the last prediction that MIR1, but not MIR2, is associated with frequent secondary rearrangements. In conclusion, the three predictions of our two MIR models were satisfied: (1) MIR2 specifically requires flanking homology on the 3′ side of the donors, but MIR1 does not (Fig. 3). (2) MIR2 requires sufficiently long displacement DNA synthesis tracts to make it partly dependent on Pol32 (Fig. 2C). (3) Additional rearrangements occur mainly through MIR1, which differs from MIR2 in that it gives rise to two single-ended DSBs in addition to the MIR product (Fig. 3).

### Analysis of secondary rearrangements in MIR1 recombinants

Finer MIR1 class analysis revealed specific associations or exclusions between additional DSB:donor SVs and donor retention (Fig. 4B; Supplemental Fig. S4). Three main classes emerged: B0, C1, and D0, accounting for 19%, 33%, and 27% of total MIR1 recombinants, respectively (67 out of 84 total, *S2* 70 bp and *S2* 1000 bp pooled) (Fig. 4A,B). High-throughput sequencing or aCGH analysis of a subset of four, six, and five such recombinants obtained with the *S2* 70-bp donor confirmed the expected chromosome II and chromosome V CNVs for each of these classes and revealed two instances of additional unselected CNVs (see below; Supplemental Fig. S5A–C). Notably, the class 1 SV [i.e., V:II(Y)] is the most common single SV observed (33 out of 38) and is primarily associated with a C donor class (C1 = 27 out of 33). Conversely, retention of the *LY* donor (i.e., B and D classes) was never associated with a V:II(Y) SV (zero out of 19 and zero out of 25, respectively) (Fig. 4B; Supplemental Fig. S4A,B). This distribution significantly departs from that of an independent assortment of *LY* donors (B0) with the II:V(Y) SV (C1), in which five out of 84 B1 events would be expected (*P* = 0.02, χ^2^ test), and indicates that the two are mutually exclusive. This observation allows mechanistic inferences as to the repair of the primary and secondary DSBs generated at donor sites following MIR1 (Fig. 4D).

Repair of the one-ended DSB generated in the *Y* region of the *LY* donor (e in Fig. 4D) upon MIR1 can occur by (1) BIR using the intact sister chromatid (f′′ in Fig. 4D), (2) BIR using the intact homolog (e′ and f′ in Fig. 4D) following additional resection and 3′ flap cleavage, or (3) gene conversion with the left side of the initiating DSB (a or b in Fig. 4D,E). Repair by BIR predicts donor class distributions starkly different from those observed (Fig. 4E). However, gene conversion followed by random segregation of the V:II(Y) translocation and the intact *LY*-containing chromatid will result in 50% of MIR1 recombinants bearing the *LY* donor (B + D class) and 50% lacking it (A + C class), not significantly different from the observed donor class distributions (52.2% and 47.8%, respectively) (Fig. 4E). Consequently, repair of the one-ended DSB generated on the internal donor predominantly occurs by gene conversion together with the *Y*-containing ends on chromosome V (Fig. 4D,E). In contrast, MIR2 recombinants exhibited the C class only, confirming that none of the terminal donors was damaged and lost in the MIR2 process.

The terminal (*S2*) donor is retained in the majority (56 out of 84) of MIR1 recombinants (C and D classes) (Fig. 4B,C). Because they are located at the same position at the *CAN1* locus on separate chromatids of the same homolog, the MIR1 translocation and the *S2* donor are not expected to cosegregate (Fig. 4D). Cosegregation thus implies that they are frequently disjoined upon interhomolog crossover or formation of an additional SV in the *CAN1*–*CEN5* interval during the repair of the initiating or secondary DSBs (Fig. 4D). For instance the V:II(Y) SV, which takes place at the *URA3* locus in the *CAN1–CEN5* interval, frequently cosegregates with the *S2* donor (C1 vs. A1 class) (Fig. 4B). The resulting 83-kb insertion of the *CAN1–URA3* interval at the *LYS2* locus leads to a duplication detected in all (five out of five) of the C1 clones analyzed by aCGH (Supplemental Fig. S5C). Consistently, careful PFGE analysis revealed the slight size difference between the resulting II:V:II chromosome (∼927 kb) and the II:V chromosome produced by MIR1 (∼915 kb), which is associated with a decreased intensity of the chromosome II band (Supplemental Fig. S3C,D). Thus, through the repair of the secondary DSB “e,” the MIR1 translocation is disjoined from *CEN5* and therefore from the *S2* donor. Our experimental setup did not allow us to quantify interhomolog crossover products resulting from repair of the initiating DSB with the chromosome V homolog as a template. However, these products were observed in half of all cases in a similar system with unrestricted homologies (Ho et al. 2010), likely explaining the proportion of D0 over B0 class (23 out of 84 and 16 out of 84, respectively).

Finally, V:II(YS2) SVs are infrequently observed (nine out of 84; SV class 2 and 3) (Fig. 4C). This SV can result from a half-crossover between the *YS*-containing initiating DSB (a or b) and the *S2* donor. The co-occurrence of V:II(YS2) and V:II(Y) SVs (class 3: three out of 84) is not significantly lower than that expected for independent occurrence of class 1 and class 2 SVs (approximately five out of 84). Consequently, the formation and segregation of these two SVs occur without detectable interference. This finding implies that cells undergoing MIR1 experience a failure to faithfully repair both initiating DSBs using the homolog as a template.

In conclusion, independent treatment of the repair of the initiating DSB and of the one-ended DSBs generated on the internal (*LY*) and terminal (*S2*) donors largely recapitulates the observed donor and SV class distributions (Fig. 4F,G). However, it fails to account for the absence or very low proportion of certain classes (A0, C0, B1, and D1), which can be explained by exclusive segregation of the LY donor with the V:II(Y) SV (Fig. 4F). With this assumption, only class A1 remained 2.6-fold lower than predicted. Our analysis thus broadly defines the mechanisms leading to the formation of secondary rearrangements initiated by a MIR1 event and can explain their segregation patterns.

### The chromosomal rearrangement capture (CR-C) assay detects MIR1 translocation with high sensitivity

**Figure 5.**
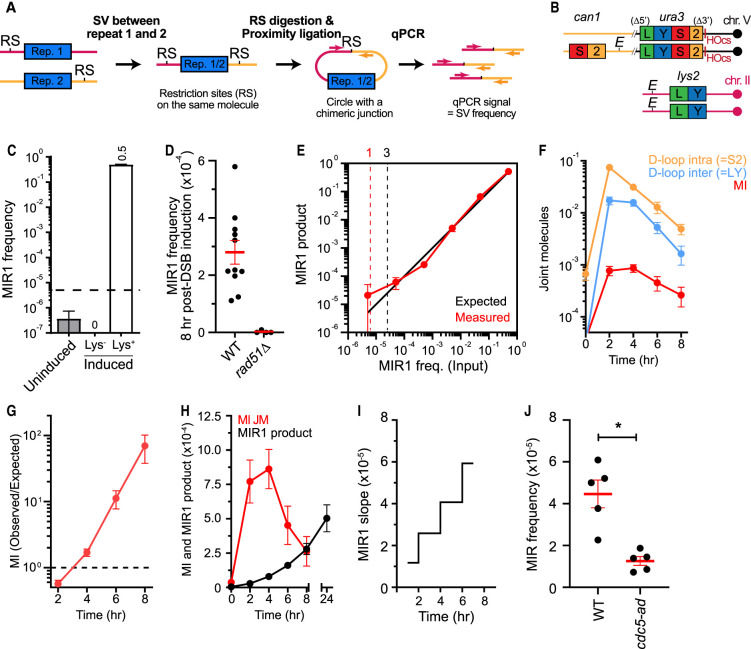
Chromosomal rearrangement capture (CR-C) reveals the late occurrence of MIR1. (*A*) Rationale of the CR-C assay for detection of repeat-mediated SVs. (*B*) Genotype of the strain used for CR-C. (*C*) MIR1 signal in a wild-type strain (APY611) either uninduced or induced without having undergone MIR (Lys^−^) and in a selected Lys^+^ translocant containing a heterozygous MIR1 translocation. (*D*) MIR1 signal 8 h after DSB induction in a wild-type strain and in a *rad51*Δ mutant (APY625 and APY704, respectively). (*E*) Experimental determination of the sensitivity of the CR-C assay. DNA from a diploid strain containing a MIR1 translocation (Lys^+^) was serially diluted in the DNA of a Lys^−^ strain (APY621), and the CR-C process was performed. Dotted lines indicate the threshold for one and three molecules expected on average per qPCR reaction. (*F*) Quantification of D-loop JMs at the *LY* and *S2* donors and of MI following DSB formation in wild-type cells (APY625). (*G*) Ratio of the observed MI over those expected based on the product of independent LY and S2 D-loops. (*H*) Kinetics of MI abundance and MIR1 translocation following DSB formation. (*I*) Slope of the MIR1 product formation curve in *H*. (*J*) MIR frequency (Lys^+^ colonies) in a wild-type strain (APY89) and *cdc5-ad* mutant (APY1483) with DSB donor configurations as in Figure 1A. (*) *P* < 0.01, two-tailed Wilcoxon test. (*E*–*G*) *n* ≥ 9 biological replicates, except for DLC at t0, which is *n* = 4.

To study the kinetics of MIR and the requirements for essential factors, we developed chromosomal rearrangement capture (CR-C), a highly sensitive molecular assay for detection of rare chromosomal translocation in a cell population (for rationale, see Fig. 5A; Supplemental Fig. S6A). CR-C relies on the proximity ligation of unique restriction sites covalently united upon translocation of the top DNA strand (Fig. 1A; for detailed experimental procedure and normalization, see the Materials and Methods) coupled with the high dynamic range of a quantitative PCR readout. We used it to detect MIR1 products in the ectopic donor configuration upon formation of a homozygous HO-induced DSB (Fig. 5B). This unrepairable system purposefully prevents growth resumption of cells repairing the break early and enables quantification of DNA JM and MIR1 product in a fixed number of cells (Piazza et al. 2019). No MIR1 product was detected prior to DSB induction or in Lys^−^ colonies recovered after DSB induction (Fig. 5C). An induced Lys^+^ colony containing a single translocation in a diploid genome exhibited the expected CR-C value of 0.5 ± 0.022 (Fig. 5C). The MIR1 product detection is Rad51-dependent, as is MIR (Fig. 5D; Piazza et al. 2017). Serial dilution of DNA from a MIR1-containing strain (induced Lys^+^) in the DNA of a MIR1-devoid strain (induced Lys^−^) showed that CR-C can reliably detect translocation frequencies down to ∼5 × 10^−5^ (Fig. 5E). Accordingly, MIR (Lys^+^) frequency and MIR1 levels per haploid genome equivalent 24 h after DSB induction were in good quantitative agreement (Supplemental Fig. S6B). The CR-C assay is thus a sensitive molecular assay for the detection of rare translocations in a cell population.

### MIR1 occurs at late time points in a subset of persisting MI JMs

The D-loop capture (DLC) (Piazza et al. 2017, 2019, 2021b; Reitz et al. 2022) and CR-C assays enabled the determination of the temporal succession of D-loops, MIs, and MIR1 product formation following DSB induction (Fig. 5F–H). Improvements to the DLC protocol further enabled estimating the absolute D-loop and MI levels per haploid genome equivalent (see the Materials and Methods; Reitz et al. 2022). D-loops at the intrachromosomal S2 donor were highest at the earliest time point assayed (2 h) and decreased monotonically twofold to threefold every 2 h (Fig. 5F). D-loops formed less frequently at the interchromosomal LY donor and remained stable between 2 and 4 h after DSB induction before declining at 6 and 8 h (Fig. 5F). The fivefold preference for intra-D-loop versus inter-D-loop formation observed at 2 h decreased to twofold afterward, indicating a kinetic delay for interchromosomal D-loop formation relative to a spatially favored intrachromosomal donor. These intrachromosomal and interchromosomal D-loop formation/decay kinetics were similar to those previously reported at these loci in haploid cells (Piazza et al. 2019, 2021a). The kinetics of MI closely matched that of the rate-limiting invasion of the interchromosomal donor (Fig. 5F). Average intrachromosomal and interchromosomal D-loops amounted to 3.1% and 1.6% of broken molecules 4 h after DSB induction. MIs formed infrequently, involving 0.086% of broken molecules. This observed quantity of MIs closely matched that expected assuming independent invasion of the intradonor and interdonor 2 and 4 h after DSB induction, with an observed/expected ratio of 0.57 and 1.69, respectively (Fig. 5G). Although MIs declined in absolute terms over time, their observed/expected ratio gradually increased past the level expected from independent invasion events (Fig. 5G). Approximately 5.4% and 16.1% of intra-D-loops and inter-D-loops, respectively, were thus part of a MI at 8 h after DSB induction. Hence, the subset of cells with JMs persisting past 4 h after DSB induction is enriched for MIs.

MIR1 occurred in a delayed fashion relative to both individual D-loops and MI formation (Fig. 5H). The increase in MIR1 products mirrored the decrease in MIs from 4 to 8 h, with little MIR1 occurring afterward (Fig. 5H). In absolute terms, the total MIR1 products observed at 8 h (2.8 × 10^−4^) amounted to only a fraction of the MIs detected at any given time point (from 8.6 × 10^−4^ at 4 h to 2.6 × 10^−4^ at 8 h) (Fig. 5H). Consequently, only a subset of MIs is converted into a MIR1 product, but this fraction increases over time, as revealed by the increasing slope of MIR1 between every time point up to 8 h (Fig. 5I). This result suggests that the pathway leading to a MIR1 product from a decreasing pool of MI intermediates is mainly active at late time points relative to DSB induction. MIR1 resolution thus broadly coincides with the timing of DNA damage checkpoint adaptation (Toczyski et al. 1997). We verified that these strains bearing two unrepairable, homozygous HO-induced DSBs do adapt (Supplemental Fig. S6C), as previously reported in similar systems in haploid and diploid cells (Toczyski et al. 1997; Galgoczy and Toczyski 2001). Consistently, MIR frequency was reduced threefold in the adaptation-deficient *cdc5-ad* mutant (Fig. 5J; Toczyski et al. 1997), with no notable difference in colony size heterogeneity (Supplemental Fig. S6D). These results suggest that checkpoint adaptation stimulates MIR.

### MIR1 does not require displacement DNA synthesis

**Figure 6.**
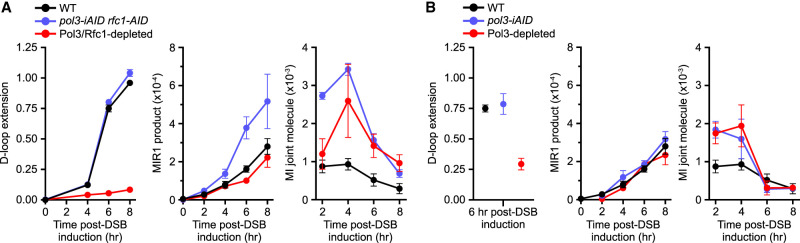
MIR1 occurs in the absence of Pol3 and Rfc1. (*A*) D-loop extension (*left*), MIR1 product formation (*middle*), and MI joint molecules (*right*) in a *pol3-iAID rfc1-9Myc-AID* strain (WDH6066) upon mock treatment or inhibitor treatment. (*B*) Same as in *A* in a *pol3-iAID* strain (WDHY6065). Data are mean ± SEM. All data are *n* ≥ 3 biological replicates, except for *pol3-iAID* CR-C and DLC, which are *n* = 2.

The translocation of the top strand by MIR1 is predicted to be achieved in an endonucleolytic fashion (Fig. 2A). Restoration of the complementary strand will involve DNA synthesis using the ssDNA as a template. Accordingly, DNA synthesis that requires displacement of a complementary strand (displacement DNA synthesis) by Pol*δ*-PCNA (Li et al. 2009; Donnianni et al. 2019) is predicted to be dispensable for MIR1 (Fig. 2A). To address this prediction, we depleted the Pol*δ* catalytic subunit Pol3 alone or in combination with the PCNA clamp loader subunit Rfc1 by coupling an auxin-induced protein degradation and a Tet-off transcriptional repression system (Tanaka et al. 2015; Donnianni et al. 2019; Kulkarni et al. 2020). As expected, depletion of Pol3 is lethal (Supplemental Fig. S7A). Addition of doxycycline and auxin resulted in rapid elimination of both proteins (Supplemental Fig. S7B,C), allowing analysis of their function specifically during DSB repair. Measurement of D-loop extension >400 bp using our D-loop extension (DLE) assay (Piazza et al. 2018) revealed that the tagged Pol3 and Rfc1 proteins did not affect recombination-associated DNA synthesis. However, codepletion of Pol3 and Rfc1 caused a >10-fold reduction in recombination-associated displacement DNA synthesis (Fig. 6A), an expected yet never demonstrated outcome. Importantly, Pol3/Rfc1 depletion did not significantly affect MIR1 product formation (Fig. 6A). Presence of the tagged proteins caused an approximately twofold increase in MIR1. JM quantification by DLC revealed that presence of either or both tags and, to a lower extent, protein depletion caused an increase in the amounts of individual D-loops and MIs (Fig. 6A; Supplemental Fig. S7D). This increase presumably results from a defect in Rfc1-dependent PCNA recruitment, which fails to direct D-loop disruption by Srs2 (Robert et al. 2006; Burkovics et al. 2013; Miura et al. 2013; Liu et al. 2017; Piazza et al. 2019). Consequently, we repeated these experiments in a *RFC1*^*+*^ strain. Depletion of Pol3 alone reduced D-loop extension by approximately threefold without substantial change to D-loops and MI levels compared with a wild-type strain (Fig. 6B; Supplemental Fig. S7D). It suggests that Rfc1, but not Pol3, tagging and depletion caused elevated JM levels in the *rfc1-AID pol3-iAID* strain. Importantly, Pol3 depletion did not affect MIR1 (Fig. 6B). These results fulfill the final prediction that MIR1 occurs in the absence of displacement DNA synthesis.

## Discussion

### Delineation of two MIR subpathways and their specific requirements

Here we provide genetic and molecular evidence for two MIR subpathways, their respective sequence and protein requirements, and their likelihood of generating SVs. MIR1 uses endonucleolytic resolution without a need for shared sequence homology between the donors (Fig. 2A). As such, it can occur between any two genomic sites and thus represents a potentially ubiquitous repeat-mediated SV formation mechanism. MIR1 results in two additional DSBs, which can be accurately repaired only if there is homology between the two donors or their surrounding regions, as is the case at allelic sites (Supplemental Fig. S2A). In the absence of such homology, there is no opportunity for accurate repair of these ends, and SVs frequently form involving the initiating and the donor sites (Fig. 4), as well as other sites exhibiting substantial levels of homology (Fig. 1). We could deduce the pathway leading to frequent secondary SVs, such as a chromosome V:II SV, produced by gene conversion or SSA between the cleavage product of the internal invasion and one of the initiating DSB ends (Fig. 4E). Finally, MIR1 occurs in the absence of Pol*δ*-PCNA, indicating that the large-scale rearrangements associated with this pathway involving multiple DNA repeats can happen without displacement DNA synthesis in an endonucleolytic fashion. This MIR1 pathway is in contrast to SV formation by repeat-mediated template switches during BIR and fork restart, which are dependent on extensive displacement DNA synthesis (Lambert et al. 2005; Malkova and Haber 2012; Marie and Symington 2022). Aborted BIR and long ssDNA accumulating during BIR progression may promote the formation of additional DNA JMs such as MIs (Štafa et al. 2014; Vasan et al. 2014; Elango et al. 2017; Zhang et al. 2023). Consequently, BIR- and SSE-mediated JM processing such as MIR may synergize to destabilize the genome.

The MIR2 pathway generates an insertion of ssDNA regions distant from the initiating DSB, between the two sites of invasion, as does MIR1 (Fig. 2B). Contrary to MIR1, however, MIR2 generates a single one-ended DSB used for product formation, precluding formation of secondary rearrangements. It uniquely requires displacement DNA synthesis and substantial sequence homology on the 3′ side of the donors relative to the orientation of the invading strand (Fig. 2B). This sequence dependency may limit its broad applicability, likely confined to allelic recombination and large segmental duplications.

### Cellular state prone to nonconservative HR outcome

Coincident HR-mediated loss-of-heterozygosity (LOH) events have recently been reported (Sampaio et al. 2020), revealing the existence of a hyperrecombinogenic state characterized by crossover resolution in a subset of a yeast population. The nature and defect of this population remain unclear. Here, we show that resolution of MI JMs into a MIR1 translocation ramps up over time, being maximal past the peak of D-loop and MI JM formation (Fig. 5H,I). This timing coincides with the onset of adaptation to the DNA damage checkpoint (Toczyski et al. 1997), at which Cdc5^PLK1^ phosphorylates a range of targets to trigger mitotic exit. Consistently, MIR is significantly reduced in an adaptation-deficient *cdc5-ad* mutant (Fig. 5J). Cdc5 targets include Mus81–Mms4 (Matos and West 2014), an SSE involved in crossover resolution (Ho et al. 2010), MIR (Piazza et al. 2017), and HR-dependent anaphase bridge cleavage in yeast and human cells (García-Luis and Machín 2014; Chan et al. 2018). Furthermore, aneuploidies of the initiating chromosomes are frequent (Fig. 1E; Piazza et al. 2017), indicating that MIR is associated with chromosome missegregation. Consequently, cells undergoing several coincidental chromosomal rearrangements may be characterized by either (1) an inability to complete HR repair or dissolve DNA JMs prior to checkpoint adaptation or (2) an inefficient activation or rapid adaptation to the DNA damage checkpoint that unleashes the activity of JM resolution enzymes. Indeed, a crippled DNA damage checkpoint promotes LOH and SV formation in *Candida glabrata*, which contributes to diversifying the genome of this quasiasexual yeast (Shor and Perlin 2021).

### Expanded sequence space for the generation of repeat-mediated SVs by HR

**Figure 7.**
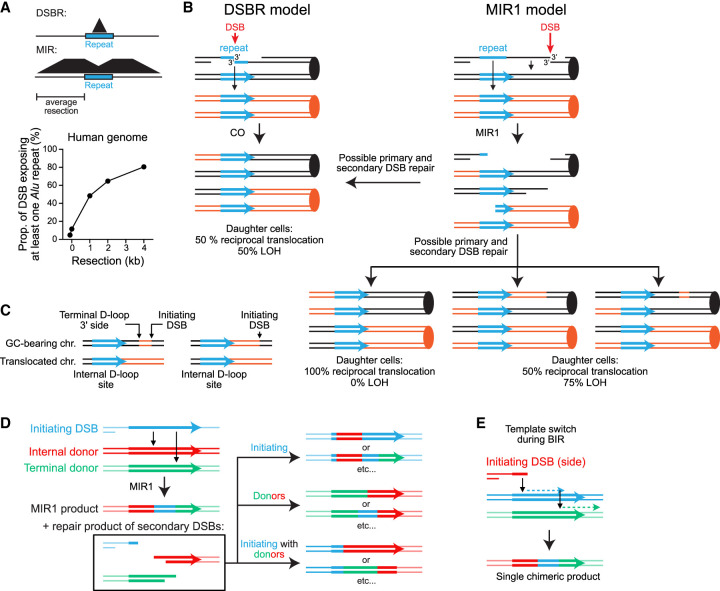
MIR and the formation of genome rearrangements. (*A*) Sequence space at risk of DSBR- and MIR-induced repeat-mediated SVs as a function of resection length. The example of *Alu* elements in the human genome is shown. (*B*) SV formation at an ectopic repeat according to the DSBR model (*left*) and MIR1 (*right*). DSBR requires the DSB and dHJs to form within the repeat, and segregation products will cause LOH or a reciprocal SV in only half of the cases. MIR1 requires the DSB to occur within the typical resection tract length from a repeat and only requires one invasion at the repeat. Depending on the relative location and orientation of the two recombining repeats, MIR1 can produce circles, inversions, and tandem duplications and deletions. Final rearrangement and gene conversion outcomes depend on the secondary DSB repair pathway, template used, and ends involved. (*C*) Repeat-mediated SV signature that can unambiguously be attributed to MIR. (*D*,*E*) MIR1 between repeats. The repair of the initiating and secondary DSBs leads to several chimeric repeats. The co-occurrence of chimeric repeats can thus be used as a discriminating signature of MIR1 relative to template switch during BIR that produces a single chimeric repeat.

The prevailing model for the formation of HR-dependent balanced SVs between two repeated genomic elements is the canonical DSB repair (DSBR) model, which entails the endonucleolytic resolution of a dHJ formed between two ectopic repeats (Szostak et al. 1983). This model assumes a DSB will form within a repeat with sufficient homology on both sides to perform DNA strand invasion and second end capture (Fig. 7A). In contrast, MIR can be initiated by repeated sequences located away from the DSB site, provided that they are exposed by resection and part of the Rad51–ssDNA filament (Fig. 7A; Piazza and Heyer 2018). Consequently, the sequence space at risk to form repeat-mediated SVs is expected to be much greater with MIR than with DSBR (Fig. 7A). Indeed, resection proceeds at ∼4 kb/h (Mimitou and Symington 2008; Zhu et al. 2008) and can span tens of kilobases in mitotically dividing *S. cerevisiae*. Hence, more than half of randomly distributed DSBs have the potential to undergo MIR after 2 h of resection despite a total repeat content of only 7% (Piazza and Heyer 2019). Likewise, resection reaches up to 3.5 kb in human U2OS cells (Zhou et al. 2014), which would make 70% of DSBs at risk of *Alu*-mediated SVs (Fig. 7A), the most frequently observed rearrangements in human tissues (Pascarella et al. 2022). This is ∼10-fold more than that expected from DSBR that requires the DSB to fall at a distance from the repeat edge (Fig. 7A). Comparatively shorter resection tracts in meiosis, averaging 0.8–1 kb both in *S. cerevisiae* and mice (Zakharyevich et al. 2010; Mimitou et al. 2017; Yamada et al. 2020), may help protect against repeat-mediated SVs by MIR. Indeed, both MIR- and DSBR-mediated crossover, but not SDSA, are highly sensitive to homology length in *S. cerevisiae*: More than 300 bp on each side of the DSB are required for detectable crossover formation (Inbar et al. 2000), and MIR drops more than proportionally with decreasing homology length (Piazza et al. 2017). This requirement for longer homology to achieve formation and/or endonucleolytic resolution of dHJ and MI JMs may mitigate the threat for genome stability posed by the most frequent short dispersed repeats such as LTR in *S. cerevisiae* and *Alu* elements in primates.

Nonetheless, rearrangement patterns in yeast consistent with MIR have been reported. For instance, a break outside a Ty element is more prone to generating rearrangements than a DSB induced within (Hoang et al. 2010). Also, the recent observation that a large proportion of recombination events induced by DSBs within LTR or Ty elements has breakpoints in unique sequences and generates frequent recombination between nontargeted repeats (Fleiss et al. 2019; Qi et al. 2023) is consistent with the induction and repair of secondary DSBs, as shown for MIR (Fig. 7B–D).

Of note, and depending on the repair of the initiating and secondary DSBs, MIR can elicit reciprocal translocations in 100% of daughter cells (Fig. 7B). Consequently, MIR1 is overall expected to lead to reciprocal translocation in >50% of cases, unlike DSBR.

### Repeat-mediated SV formation in humans and sequence signatures of MIR

Over the last decade, long-read sequencing technologies have allowed high-confidence detection of balanced SVs in the human genome (Logsdon et al. 2020). A comparative analysis of multiple sequencing methodologies estimated that long-read sequencing detects sevenfold more variation in the form of insertions/deletions (indels) and SVs relative to high-coverage Illumina whole-genome sequencing (Chaisson et al. 2019). Repeated elements, particularly *LINE* and *Alu* repeats, appear to be one of the primary sources of these de novo SVs, which are surprisingly common even in healthy individuals (Chaisson et al. 2019; Balachandran et al. 2022; Pascarella et al. 2022). However, much remains unexplored as to how repeat-mediated SVs contribute to human health and disease and which specific HR subpathways are involved in their formation; namely, DSBR, BIR, SSA, and MIR.

Here, we aimed to provide sequence signatures that unambiguously distinguish SVs produced by MIR from those produced by other HR pathways (Fig. 7C–E). Indeed, repair of the secondary DSBs produced by MIR1 can lead to a reciprocal translocation at the repeat involved in MIR1 accompanied by a gene conversion of the intervening sequence between the repeat and the initiating DSB (Fig. 7B). In a subset of daughter cells, this repair outcome will lead to the association of a translocation on one chromosome and the presence of a flanking LOH on another chromosome (Fig. 7B,C). Of note, both arise from the repair of secondary DSBs. The repeat-distal edge of the LOH tract reveals the position of the initiating DSB (Fig. 7C). If the LOH tract does not extend to the repeat, the repeat-proximal edge of the LOH indicates the approximate 3′ side of the cleaved terminal D-loop (Fig. 7C). To our knowledge, no mechanism other than MIR1 can produce such matched SV/LOH tracts at and flanking a repeated sequence.

In addition, MIR1 can recombine long repeats such as *LINE*s, inserting the sequence of the repeat flanking the initiating DSB between the translocated repeats (Fig. 7D). In a subset of cases, the initiating and secondary breaks will be repaired using the chimeric SV repeat, leading to the presence of two chimeric repeats (Fig. 7D). The presence of these matched chimeric repeats can distinguish MIR1 from a template switch event during BIR, which is expected to produce only one chimeric repeat (Fig. 7E). Such signatures may help to refine mechanistic inferences made from long-read sequencing data and probe the associated genetic defects.

## Materials and methods

### Diploid *S. cerevisiae* strains and genetic constructions.

The genotype of the diploid *S. cerevisiae* strains (W303 *RAD5*^*+*^ background) used in this study are listed in Supplemental Table S1. Diploidy buffered for rearrangements that may cause loss of essential genes and allowed for capture of more chromosomal rearrangements. Most genetic constructs have already been described in Piazza et al. (2017) and are available as GenBank files in the Supplemental Data Set S1. Briefly, strains contained a heterozygous copy of the *HO* endonuclease gene under the control of the *GAL1/10* promoter at the *TRP1* locus on chromosme IV. The DSB-inducible construct contained the 117-bp HOcs (Fishman-Lobell and Haber 1992), various possible fragments of the *LYS2* gene lacking a start and a stop codon, and a unique 453-bp-long sequence derived from the PhiX genome. The DSB-inducible construct replaced the *URA3* locus (−16 to +855 from the start codon) on chromosome V. The HOcs at the mating type loci (*MAT*) on chromosome III was inactivated by a point mutation to prevent HO cleavage (*MAT***a**-inc*/MAT****α***-inc) (Fishman-Lobell and Haber 1992). In the allelic interchromosomal donor configuration, the *LY* and *S2* donors replaced the original *LYS2* ORF on chromosome II. In the ectopic *cis* and *trans* donor configurations, the *S2* donor and its 70-, 500-, or 1000-bp-long downstream sequence were inserted at the constitutively mutated *can1-100* locus, which caused a deletion of the beginning of the gene (−342 to +439 bp from the start codon). *S2* was oriented so as to avoid generating a dicentric II:V chromosome upon translocation with the *LY* donor left on chromsome II.

The *RFC1-AID-9Myc::hphMX* construct was a gift from Neil Hunter and has been described previously (Kulkarni et al. 2020). The *POL3-iAID* construct was a gift from Lorraine Symington and has been described previously (Tanaka et al. 2015; Donnianni et al. 2019). The constructs for *TIR1* and *TetR* expression integrated at *SSN6* have been described previously (Tanaka et al. 2015). Introduction of the *cdc5-ad* mutation (*T751G*) (described in Toczyski et al. 1997) at its endogeneous locus was achieved by CRISPR/Cas9-mediated gene editing by targeting the sequence 716–735 bp from the start codon (5′-GTAATTAGGTGTTCCGCATA**TGG**-3′; PAM in bold) and providing the dsDNA repair template (top strand): 5′-GTGAACGTAAATACACAATATGCGGAACACCTAATTACATCGCACCTGAAGTGTGGATGGGTAAGCATTCTGGACATTCATTTGAAGTAG-3′ (nucleotide substitutions are underlined). The template contained a PAM-inactivating silent mutation in addition to the *T751G* mutation.

### Media and culture conditions

Synthetic dropout (SD) and rich YPD (1% yeast extract, 2% peptone, 2% dextrose) solid and liquid media were prepared according to standard protocols (Treco and Lundblad 2001). Liquid YEP-lactate (1% yeast extract, 2% peptone, 2% lactate) was made using 60% sodium DL-lactate syrup. All cultures were performed at 30°C.

### Pol3 and Rfc1 depletion using the auxin-inducible degron system

For time courses involving *pol3-iAID RFC1* or *pol3-iAID rfc1-AID-9Myc* strains, a single colony of the appropriate strain was used to inoculate a 5-mL YPD culture, grown to saturation overnight, and diluted in 250 mL of YEP-lactate. Following ∼13–16 h of growth overnight at 30°C, the OD_600_ of the culture was determined, and the culture was split equally into two smaller flasks. Protein depletion was conducted as in Donnianni et al. (2019) with minor modifications (Supplemental Fig. S7B). Doxycycline (0.1 µg/mL) was added to the “with inhibitor” culture. One hour later, the culture was supplemented with 50 µg/mL doxycycline and 1.5 mM indole-3-acetic acid (IAA) (2.5 mM in Donnianni et al. 2019). An equal volume and concentration of solvent was added to the “without inhibitor” culture at both times (Donnianni et al. 2019). One hour later, the DSB was induced by addition of 2% galactose. Doxycycline and IAA were prepared as described in Tanaka et al. (2015).

### Lysine prototrophy-based translocation assay in *S. cerevisiae*.

The translocation assay has been described previously upon HO induction in liquid media (Piazza et al. 2017, 2021b) or on plates. Briefly, the basal Lys^+^ frequency was determined by plating yeast cells exponentially growing in YEP-lactate liquid culture on SD-LYS and YPD plates (control plating). The expression of the HO endonuclease was triggered in the remaining liquid culture upon addition of 2% galactose. Two hours after galactose addition, when HO cutting was >99% (Piazza et al. 2017, 2021b), the induced Lys^+^ frequency was determined by plating again on SD-LYS and YPD plates. Basal and induced Lys^+^ frequencies as well as viability were determined after incubation of the plates for 2–3 d at 30°C. Alternatively, HO expression was induced by plating an exponentially growing culture on YEP-lactate on YEP and SD-LYS plates containing galactose, which allowed us to determine viability and Lys^+^ frequency upon DSB formation, respectively. The basal viability and Lys^+^ frequency in the absence of DSB formation were determined upon plating cells on glucose-containing YPD and SD-LYS plates. Both protocols yielded similar MIR frequencies. At least three independent replicates were performed for each strain. Translocation frequencies are reported in Supplemental Table S2.

### Microcolony formation assay

The ability to divide following formation of one repairable (heterozygous) and two unrepairable (homozygous) DSBs was determined upon plating exponentially growing YP-lactate cultures on galactose-containing plates, incubating them for 16 h at 30°C, and counting the number of microcolonies (defined as more than six cell bodies) under the microscope, similar to Sandell and Zakian (1993) and Toczyski et al. (1997).

### Dilution series

Cell viability was assessed on YPD + solvent or YPD + 1.5 mM IAA and 50 µg/mL doxycycline as follows: Five-milliliter YPD cultures in 15-mL glass tubes were inoculated with a single colony corresponding to the appropriate strain and grown on a rotator overnight at 30°C. After ∼16 h of growth, the OD_600_ of the cultures was determined, and the cultures were used to inoculate fresh 5-mL YPD cultures at an equivalent OD. The 5-mL cultures were grown on a rotator for ∼6 h at 30°C, the OD_600_ of the cultures was determined again, and all cultures were diluted to an OD_600_ of 0.19. A series of 1:10 dilutions was prepared for each of the strains from these starting dilutions and spotted onto YPD + solvent or YPD + 1.5 mM IAA and 50 µg/mL doxycycline plates in parallel using a multichannel pipette. Plates were imaged following 2 d of growth at 30°C.

### Southern blot analysis of the Lys^+^ recombinants

Independent Lys^+^ colonies were patched on SD-LYS plates, and their DNA was extracted from a 5-mL SD-LYS liquid culture saturated overnight. DNA was digested by HindIII (for the interchromosomal donor construct), PstI (for the ectopic *S2* 70-bp donor construct), or PstI + EcoRI (for the ectopic *S2* 1000-bp donor construct) for 4 h at 37°C and migrated overnight in 0.8% agarose-LE (Affymetrix) in 1× TBE at 50 V. The DNA was transferred from the gel onto an Amersham Hybond-XL membrane (GE healthcare) following the manufacturer's instructions (alkali protocol). The membrane was blocked with Church buffer (1% BSA, 0.25 M Na_2_HPO_4_ at pH 7.3, 7% SDS, 1 mM EDTA) for 2–3 h at 65°C. The *LY*, *S2*, or *LYS2* probes (2, 2, and 4 kb long, respectively), together with phage λ DNA (molecular ladder), were radiolabeled by random priming with 6000 Ci/mmol P^32^-*α*dCTP (Perkin-Elmer) using the Decaprime II kit (Ambion, Inc.) and incubated with the membrane overnight at 65°C. After three to five washes for 10 min at 65°C (20 mM Na_2_HPO_4_ at pH 7.3, 1% SDS, 1 mM EDTA), membranes were exposed for 8–24 h, and the storage phosphor screen (GE healthcare) was scanned on a Storm phosphorimager (Molecular Dynamics).

### Molecular karyotyping analyses

Preparation of DNA plugs and pulse-field gel electrophoresis (PFGE) of Lys^+^ recombinants were performed as described previously (Piazza et al. 2017). Array comparative genomic hybridization (aCGH) copy number profiling and characterization of rearrangement junctions through Oxford nanopore long-read sequencing were carried out following procedures described previously (Zhang et al. 2013; Heasley et al. 2020). Whole-genome sequencing was carried out as follows: Barcode-indexed sequencing libraries were generated from 250 ng of each genomic DNA sample. Samples were RNase A-digested (NEB) and sheared on an E220 focused ultrasonicator (Covaris). The samples were size-selected for fragment sizes of 480–600 bp on a Pippin-HT instrument (Sage Science). The samples were then converted into sequencing libraries using a Kapa DNA Hyper library preparation kit (Kapa Biosystems-Roche). The libraries were amplified with 13 PCR cycles, analyzed with a Bioanalyzer 2100 instrument (Agilent), quantified by fluorometry on a Qubit instrument (LifeTechnologies), and combined at equimolar ratios. The pool was quantified by qPCR with a Kapa library Quant kit (Kapa Biosystems-Roche) and sequenced on an Illumina MiSeq with paired-end 300-bp reads. Trimmed reads were aligned to the reference *S. cerevisiae* S288c R64-2-1 genome with Bowtie 2 (Langmead and Salzberg 2012) with default parameters.

### D-loop capture (DLC) and D-loop extension (DLE) assays

The DLC and DLE assays have been described previously (Piazza et al. 2018, 2019; for a step-by-step protocol, see Reitz et al. 2022). A psoralen cross-link reversal step adapted from Yeung et al. (1988) was added prior to the qPCR step, which removed various amplification biases and enabled quantitative comparison of the amount of distinct JMs (Reitz et al. 2022). Briefly, psoralen cross-link reversal was performed upon incubation of purified DNA in decross-linking solution (100 mM KOH, 10 mM Tris-HCl at pH 8.0, 1 mM EDTA) for 30 min at 90°C. pH was neutralized by addition of 73 mM Na-acetate, and the DNA was used for qPCR quantification at a 1/10 final concentration. Primers used are listed in Supplemental Table S3.

### Chromosomal rearrangement capture (CR-C) assay

Genomic DNA was purified from 5 × 10^8^ cells collected at various times after DSB induction by spheroplasting cells with zymolyase, protein digestion, phenol–chloroform–isoamyl alcohol (25:24:1) extraction, isopropanol precipitation, and RNase A treatment according to standard protocols. DNA was quantified on a Life Technologies Qubit 2.0 fluorometer using the dsDNA HS assay kit (Invitrogen Q32854) according to the manufacturer's instructions. Five-hundred nanogreams of DNA was digested for 1 h at 37°C with 50 U of EcoRI-HF (NEB R3101), and the enzyme was inactivated for 20 min at 65°C. Eighty nanograms of digested DNA was ligated in 800 µL of ligation buffer (50 mM Tris-HCl at pH 8.0, 10 mM MgCl_2_, 10 mM DTT, 100 ng/µL BSA,1 mM ATP) with 20 U of DNA ligase T4 (NEB M0202) for 1.5 h at 16°C. DNA was subsequently purified by phenol:chloroform:isoamyl alcohol (25:24:1; Sigma-Aldrich P3803) extraction followed by isopropanol precipitation. DNA was resuspended in 40 µL of TE (pH 8; 10 mM Tris-HCl, 1 mM EDTA), and 2 µL was used per 20 µL of qPCR reaction, representing ∼10^5^ haploid genomes per reaction. Single-step qPCR amplification was performed on a Roche LightCycler 480 or a Bio-Rad CFX96 thermocycler using the respective manufacturer's SYBR Green kit and instructions. The amplification of the rearranged molecule produced upon circularization of the 4628-bp MIR1-containing DNA fragment was normalized on the average circularization efficiency of three DNA fragments of similar size (4327, 4701, and 4888 bp). Primers used are listed in Supplemental Table S3.

### Immunoblotting

Protein extracts for Western blot were prepared using a standard protocol for tricholoroacetic acid (TCA) extraction. An ∼25-µL sample was run out on a 7.5% SDS-PA at 150 V for ∼1 h alongside a protein standard (Bio-Rad Precision Plus protein dual-color standard). Samples were transferred to a PVDF membrane (Bio-Rad TransBlot Turbo midsize LF PVDF membrane) using the Bio-Rad Trans-Blot Turbo transfer system. The membrane was then blotted using mouse antimini-AID tag (1:1000) primary antibody (MBL M214-3), mouse anti-c-Myc 9E11 (1:1000) primary antibody (Santa Cruz Biotechnology sc-47694), mouse anti-GAPDH (1:10,000) primary antibody (Thermo Fisher MA5-15738), and antimouse HRP-conjugated (1:1000) secondary antibody (Agilent P0447).

### Calculation of the proportion of repeats at risk of SV formation by DSBR or MIR

The RepeatMasker BED track for the human genome assembly hg38 T2T CHM13v2.0 was obtained from the UCSC table browser on October 19, 2022. Lines corresponding to *Alu* elements were retained and sorted with bedtools sort, and overlapping elements were merged with the bedtools merge function, providing the proportion of the genome made of *Alu* elements (7%). Intervals were extended by 1, 2, and 4 kb and merged, and the genome fraction at risk of *Alu*-mediated SVs through MIR was computed. Alternatively, initial intervals were shortened by 0.1 kb on each side to determine the genome fraction at risk of *Alu*-mediated SVs through DSBR, which required the DSB to fall away from the repeat element edges.

### Data availability

Raw sequencing data have been deposited at SRA BioProject PRJNA967211. Processed data (coverage of Illumina reads) aligned on the reference R64-2-1 *S. cerevisiae* genome, raw aCGH data, and SV-supporting nanopore reads have been deposited at GEO projects GSE232732 and GSE233144. A summary of data availability for MIR samples is provided in Supplemental Table S4.

## Supplementary Material

Supplement 1

Supplement 2

Supplement 3

Supplement 4

Supplement 5

Supplement 6

Supplement 7

Supplement 8

Supplement 9

Supplement 10

Supplement 11

Supplement 12

Supplement 13
