# Supplementary figures and images for: Delineation of two multi-invasion-induced rearrangement pathways that differently affect genome stability
Source: Genes Dev. 2023 Jul 1;37(13-14):621–39. doi: 10.1101/gad.350618.123 (PMC10499017; doi:10.1101/gad.350618.123)

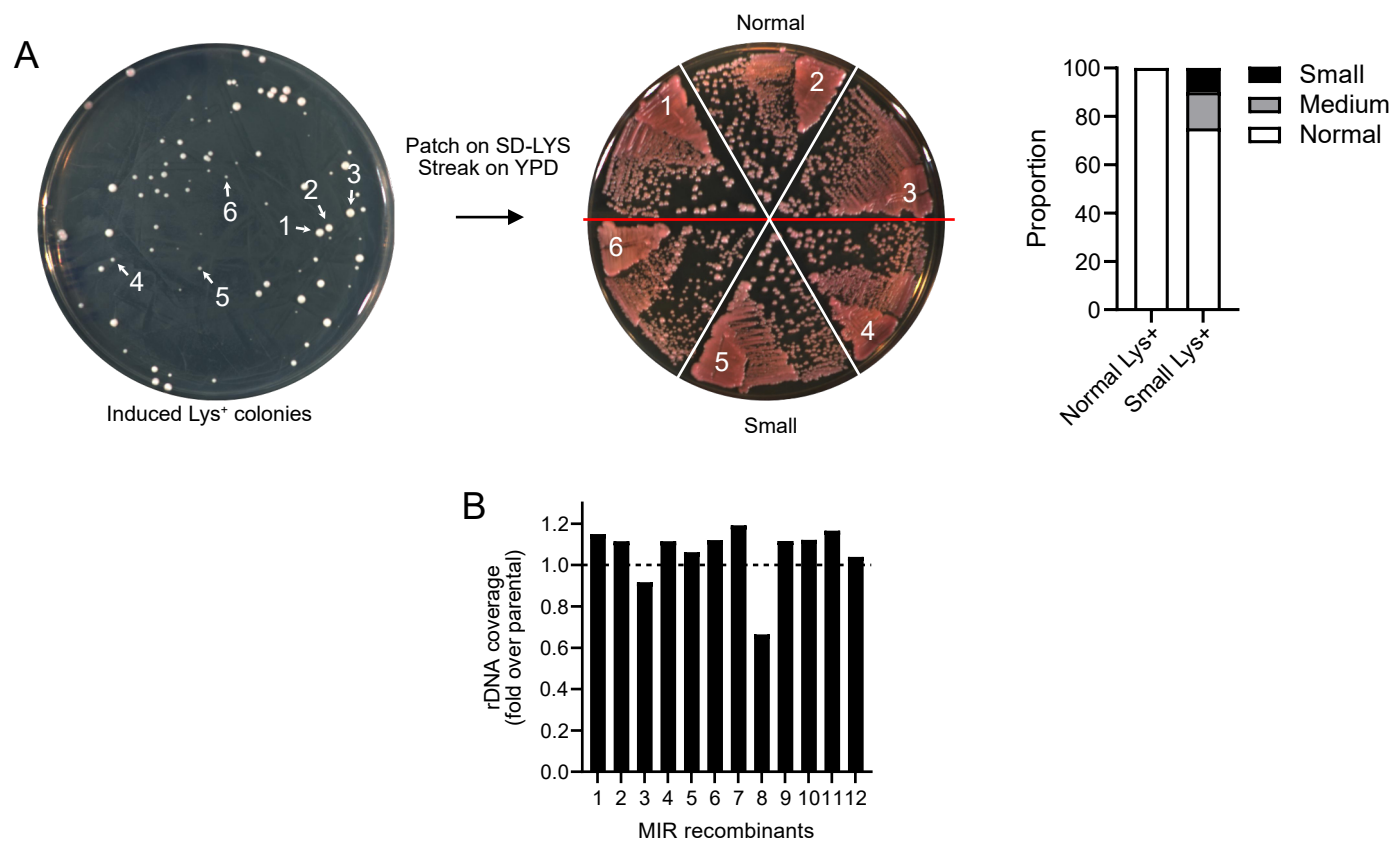

Figure S1

Supplement: Supplement 1 [file SupplementalFigS1.pdf]

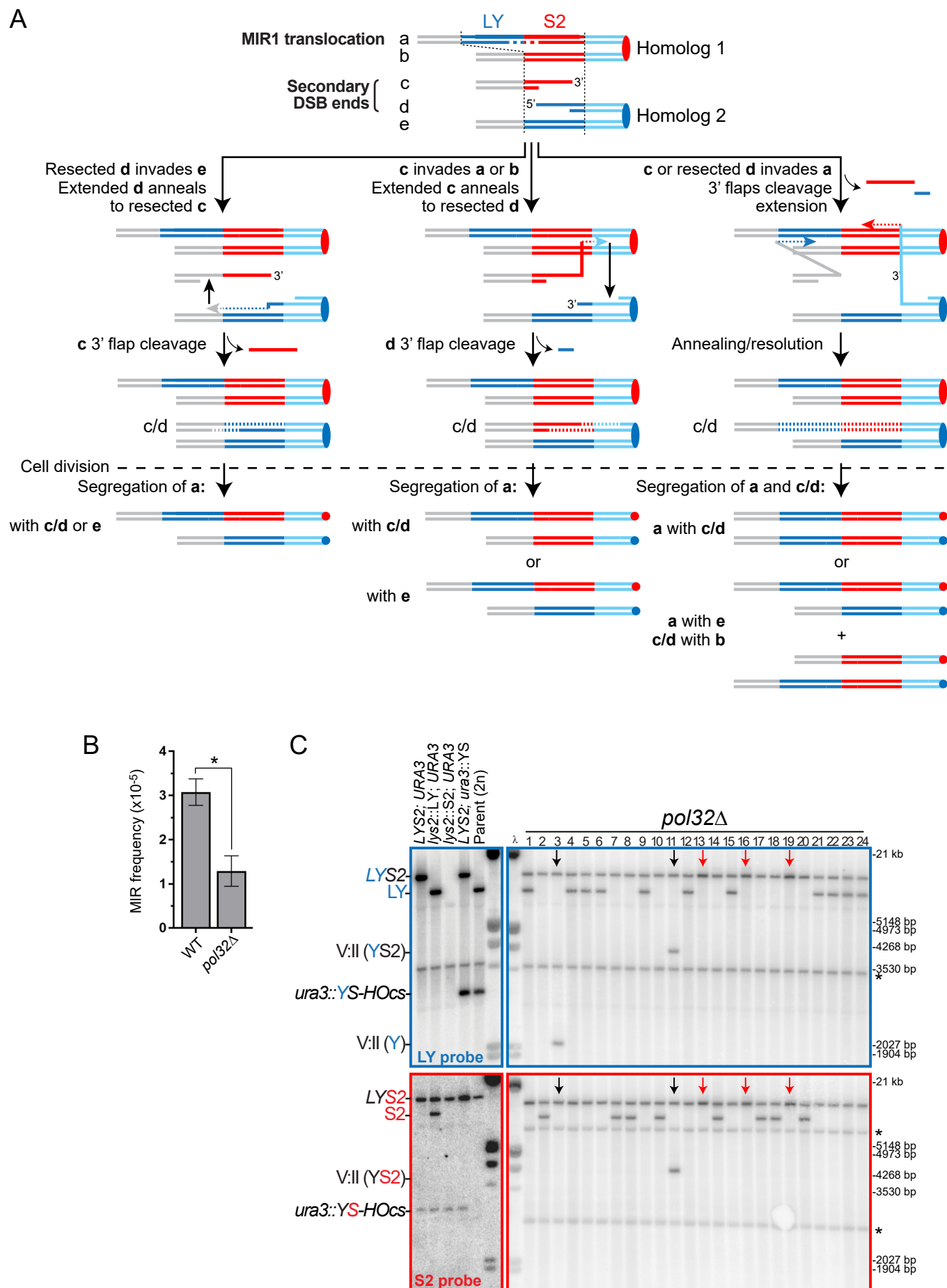

Supplement: Supplement 2 [file SupplementalFigS2.pdf]

A

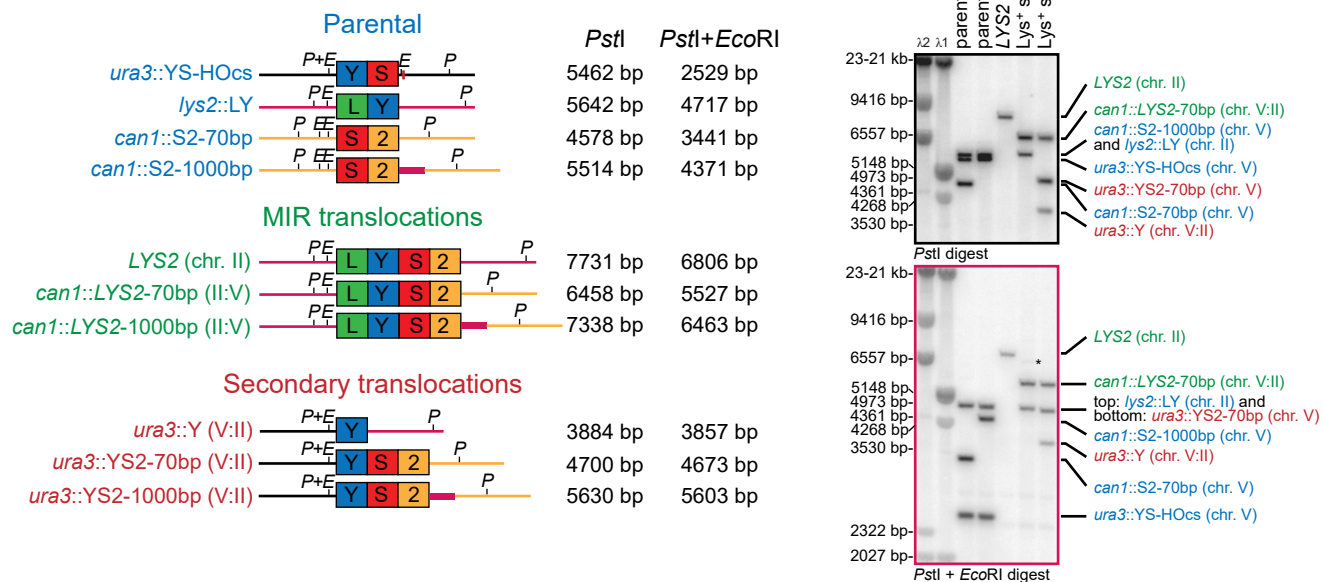

B

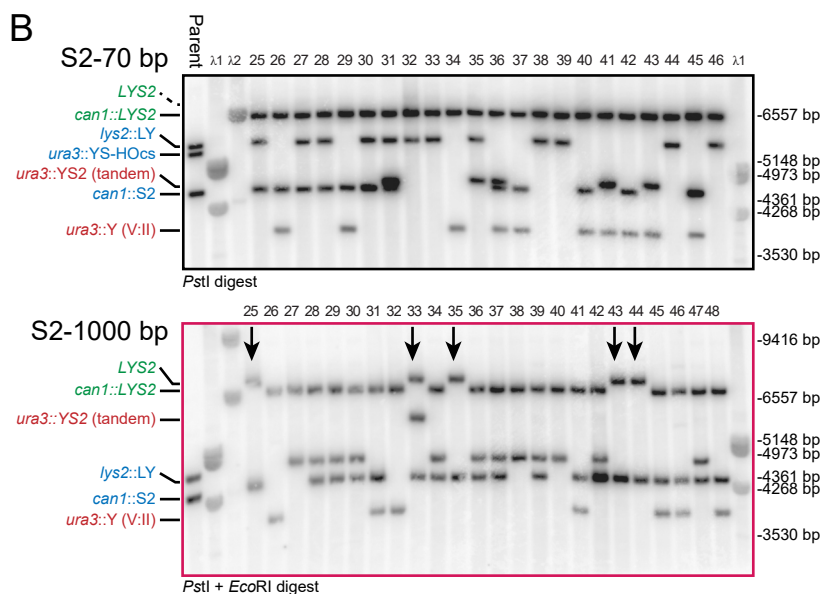

C

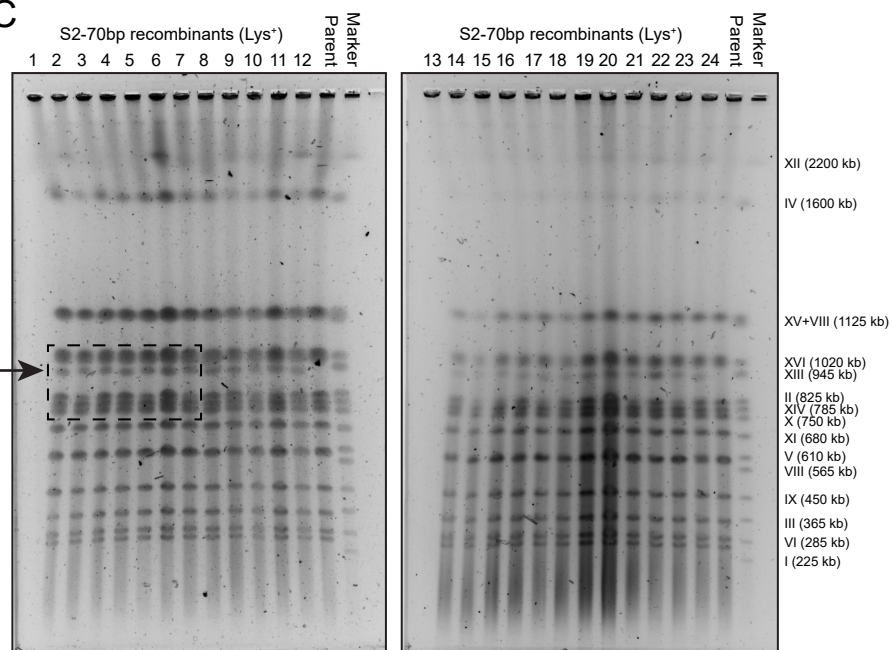

D

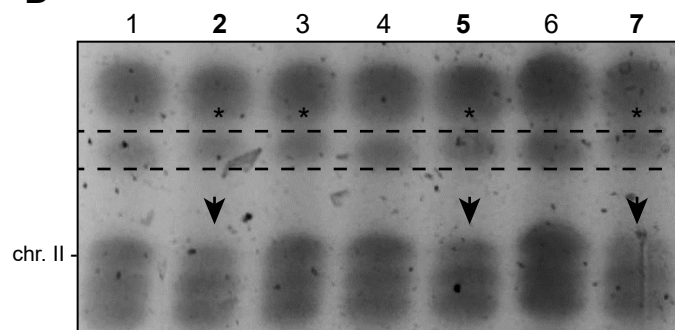

Figure S3

Supplement: Supplement 3 [file SupplementalFigS3.pdf]

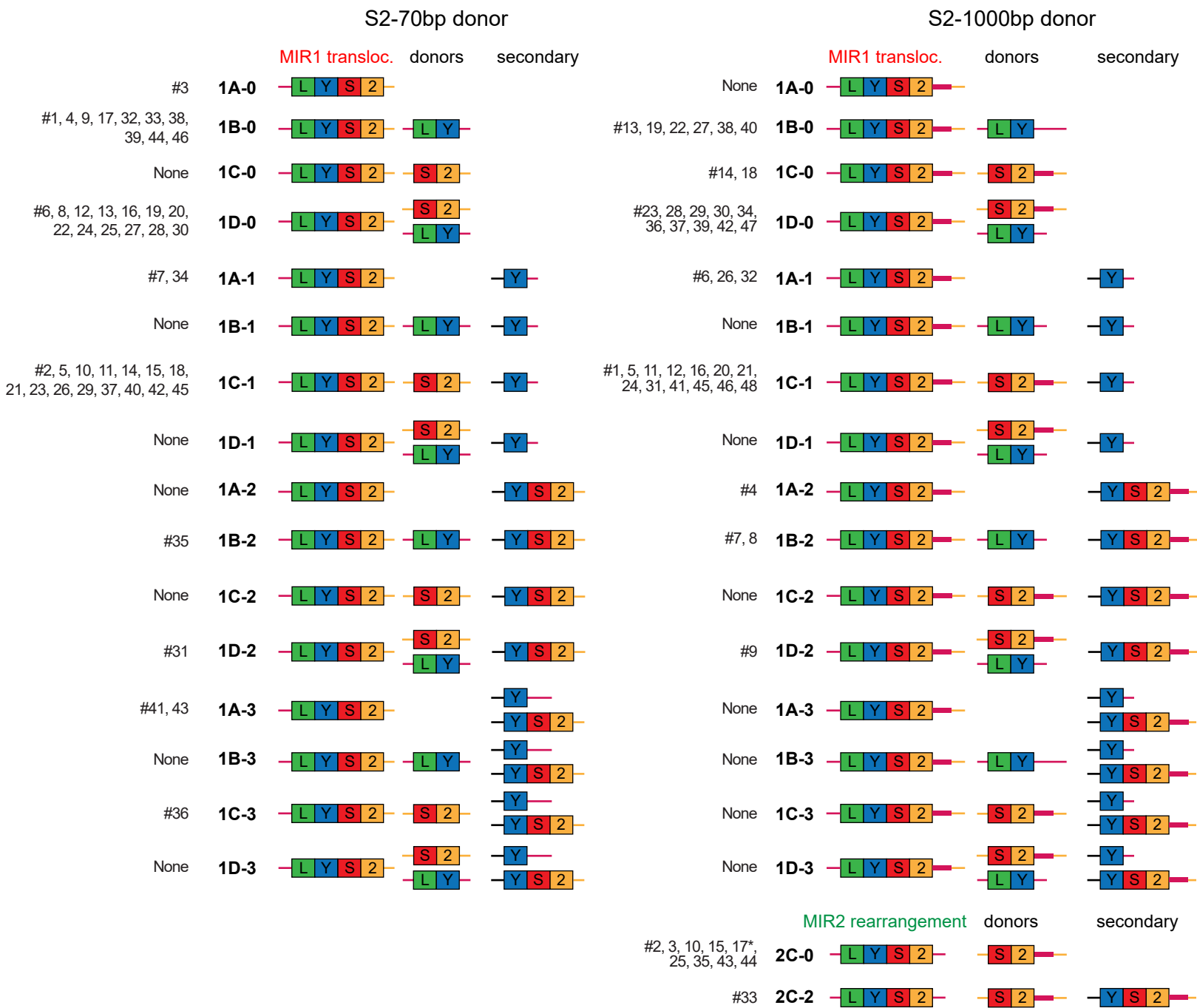

Figure S4

Supplement: Supplement 4 [file SupplementalFigS4.pdf]

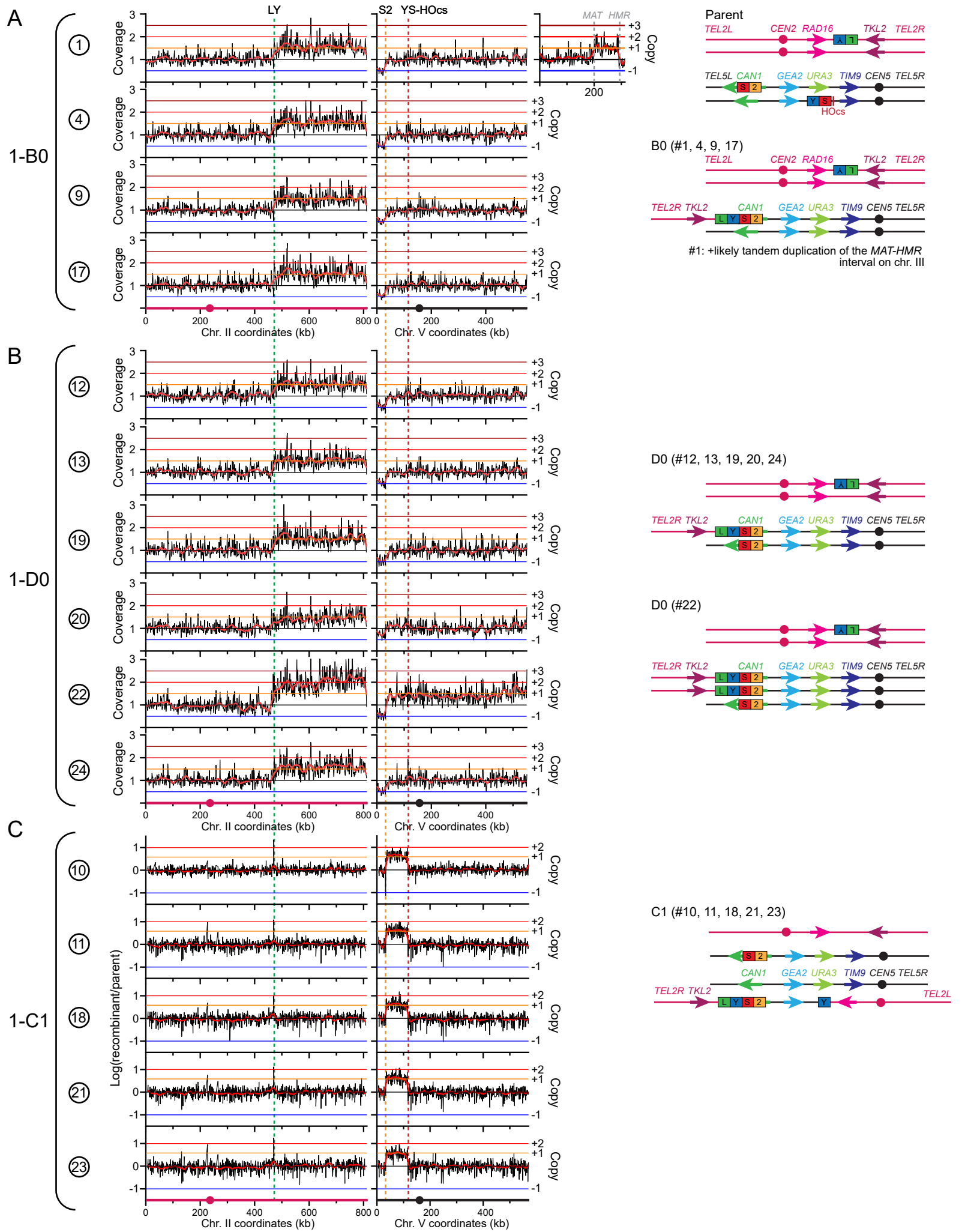

Figure S5

Supplement: Supplement 5 [file SupplementalFigS5.pdf]

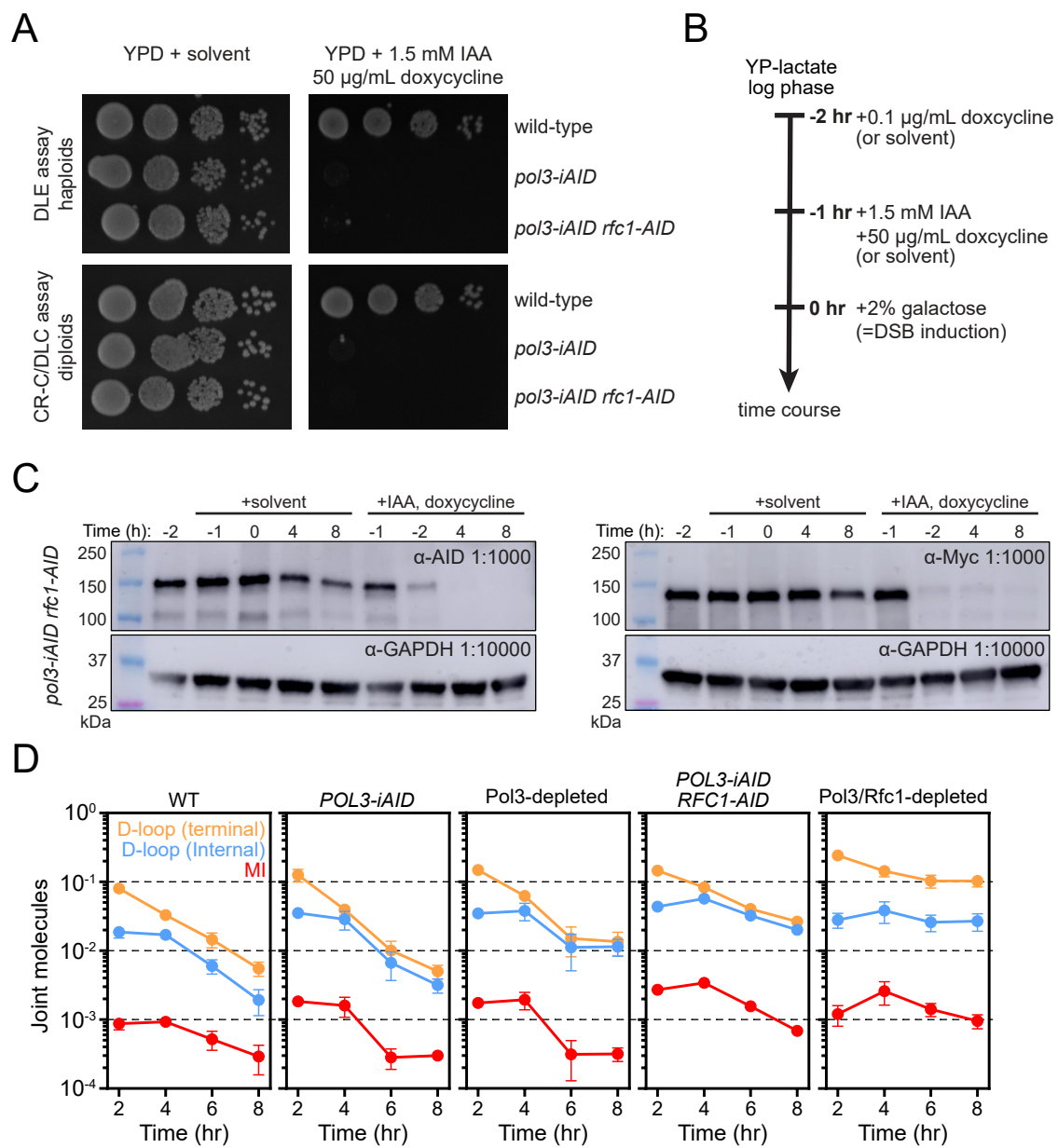

Figure S7

Supplement: Supplement 7 [file SupplementalFigS7.pdf]
