# Supplementary material for: Delineation of two multi-invasion-induced rearrangement pathways that differently affect genome stability
Source: Genes Dev. 2023 Jul 1;37(13-14):621–39. doi: 10.1101/gad.350618.123 (PMC10499017; doi:10.1101/gad.350618.123)
Supplement: Supplement 12 [file Supplemental.docx]

# Supplementary information

# Supplementary figure and table legends

**Figure S1** (related to Figure 1): **A**) Normal and small colonies on the selection plate exhibit similar colony sizes upon re-streaking. Left: representative plates. Right: quantification of the growth of 20 normal and small Lys^+^ colonies. **B**) Quantification of rDNA coverage, normalized onto the average genome-wide coverage and presented relative to the rDNA coverage in the parental strain (WDHY4260/APY89).

**Figure S2** (related to Figure 2): (A) Model of secondary DSB repair following MIR and expected segregation outcomes. (B) MIR translocation frequency in wild-type and *pol32Δ* strains (WDHY4260 and WDHY4408, respectively; data from Piazza *et al.* 2017). (C) Southern blot analysis of 24 independent Lys^+^ recombinants obtained in a *pol32Δ* mutant. Arrows indicate an anomaly in the segregation pattern, either because of additional rearrangements (black arrows) or presence of two *LYS2* translocation (red arrows; band intensity is two-fold higher than in other lanes). * indicates non-specific hybridization.

**Figure S3** (related to Figure 2): (A) Structure, predicted length, and Southern blot validation of the DSB-inducible and donor substrates (blue), MIR translocation products (green) and secondary rearrangements products (red). (B) Southern blot analysis of recombinants #25-46 and #25-48 obtained with either limited (*S2*-70bp) or extensive (*S2*-1000bp) 3’ flanking homologies, respectively. (C-D) PFGE analysis of recombinants #1-12 obtained with the *S2*-70bp and *S2*-1000bp donors, respectively. The expected size of the II:V chromosome resulting from the MIR translocation is ~915 kb based on the W303 reference genome (890 kb in S288c). The inset shows subtle size difference for the neo-chromosome, with higher migrating species associated with a decrease of chr. II signal (arrows).

**Figure S4** (related to Figures 3 and 4): Structure of the MIR1 and MIR2 recombinants deduced from the Southern blot analysis with *S2*-70bp and *S2*-1000bp donors. * marks a composite strain bearing both a MIR1 and MIR2 product.

**Figure S5** (related to Figures 3 and 4): (A-C) Copy number analysis of four MIR1 recombinants obtained with the *S2*-70bp donor belonging to the B0 class (A), the D0 class (B), and the C1 class (C), and their deduced genomic structure. Recombinant #1 exhibited an additional unselected CNV spanning the *MAT-HMR* interval. Recombinant #22 exhibited two copies of the II:V translocated chromosome. Copy number profiles were obtained from paired-end sequencing data (A, B) or aCGH (C).

**Figure S6** (related to Figure 5): (A) Strain and procedure for MIR1 quantification by CR-C. The parental strain (APY625) contains a homozygous DSB-inducible construct at the *ura3* locus on chr. V, which purposefully precludes repair and growth resumption. It bears ~2 kb homology with ectopic donors: a homozygous *LY* donor at the *lys2* locus on chr. II and a heterozygous *S2* donor at *can1* on chr. V. A 152 bp unique sequence containing an *Eco*RI site was introduced 3’ of the *S2*-70bp donor. (B) Comparison of induced MIR frequencies in wild-type cells determined genetically (*i.e.* Lys^+^ colonies), and molecularly by CR-Capture 24 hours post-DSB induction. Parental strains differ in the number of DSB: MIR frequencies were obtained from a strain containing a heterozygous, repairable DSB site (APY611), while CR-Capture frequencies originated from a homozygous, unrepairable DSB site (APY625). * indicates that MIR values were divided by two in order to obtain a frequency per haploid genome equivalent. (C) Formation of microcolonies (>6 cell bodies) after 16 hours of YP-Gal plating of exponentially growing cells either lacking a HOcs (APY621), bearing one heterozygous (*i.e.* repairable on the homolog) HOcs, or bearing two homozygous (*i.e.* unrepairable) HOcs at the *ura3* locus on chr. V in WT (APY89 and APY625, respectively) or *cdc5-ad* (APY1483 and APY1484, respectively) diploid strains. Cells containing unrepairable DSBs only produce 20% microcolonies in a checkpoint adaptation-dependent manner. Dot: biological replicate. Line: median. (D) Colony size heterogeneity in MIR translocants is similar in WT (APY89) and *cdc5-ad* (APY1483) strains.

**Figure S7** (related to Figure 6): (A) Viability of wild-type, *pol3-iAID*, and *pol3-iAID rfc1-9Myc-AID* haploids used for DLE (APY266, WDHY6053, WDHY5067) and diploids used for CR-C (APY625, WDHY6065, WDHY6066) in rich media or inhibitor-containing media. Each spot corresponds to a 10-fold dilution. (B) Experimental scheme for Pol3 and Rfc1 depletion and DSB induction. (C) Western blot of untreated and inhibitor treated wild-type, *pol3-iAID*, and *pol3-iAID rfc1-9Myc-AID* haploids revealed with an anti-AID antibody (left; both proteins) and an anti-Myc antibody (right; Rfc1). Pol3-AID and Rfc1-9Myc-AID migrate at the same position. Time is indicated relative to DSB induction. (D) Absolute joint molecules detected by DLC in a wild-type strain (APY625, n≥8), in a *pol3-iAID* strain with and without inhibitors (WDHY6065, n=2), and in a *pol3-iAID rfc1-AID-9Myc* strain with and without inhibitor (WDHY6066, n=3).

**Table S1: Relevant genotype of *Saccharomyces cerevisiae* strains used in this study**

**Table S2: Induced Lys^+^ frequencies**

Sheet 1: Induction in liquid

Sheet 2: Induction on plate

**Table S3: Primers used in this study for the DLC, DLE, and CR-C assay.**

Sheet 1: DLC primers

Sheet 2: DLE primers

Sheet 3: CR-C primers

**Table S4: Molecular karyotyping summary and data availability.**

# Supplementary Dataset S1

Annotated sequences of the genetic constructs used in this study in Genbank format.
