# Supplementary material for: Delineation of two multi-invasion-induced rearrangement pathways that differently affect genome stability
Source: Genes Dev. 2023 Jul 1;37(13-14):621–39. doi: 10.1101/gad.350618.123 (PMC10499017; doi:10.1101/gad.350618.123)
Supplement: Supplement 6 [file SupplementalFigS6.pdf]

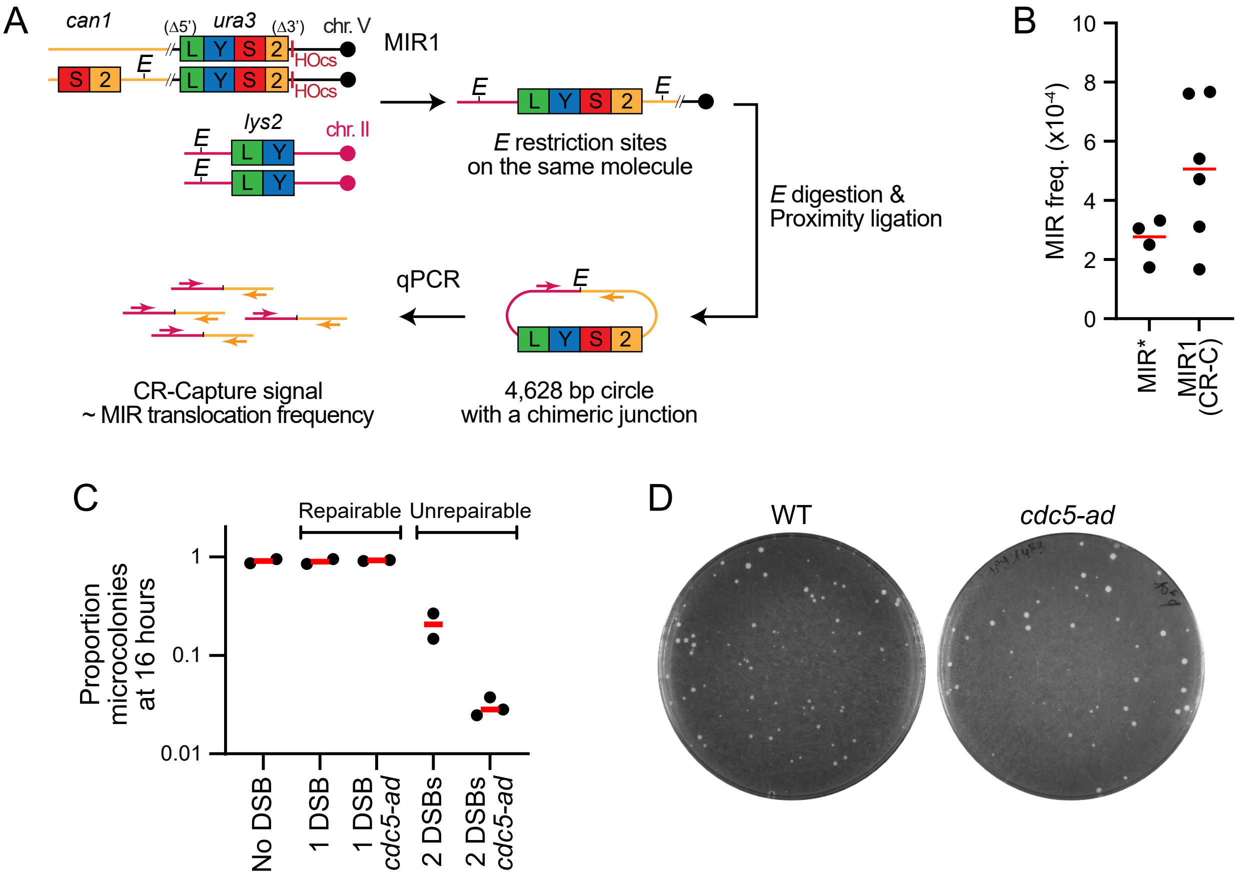
A
can1
(Δ5')
ura3
(Δ3')
chr. V
MIR1
HOcs
E
lys2
chr. II
E restriction sites
on the same molecule
E digestion &
Proximity ligation
qPCR
CR-Capture signal
~ MIR translocation frequency
4,628 bp circle
with a chimeric junction
B
MIR freq. (x10-4)
MIR*
MIR1 (CR-C)
C
Proportion microcolonies at 16 hours
Repairable
Unrepairable
No DSB
1 DSB
1 DSB cdc5-ad
2 DSBs
2 DSBs cdc5-ad
D
WT
cdc5-ad

Figure S6
